# Low SLC8A1 in Colorectal Cancer Dictates Radiotherapy Resistance Through Calcium‐Dependent ANXA2^+^sEVs Driving M2 Polarization of TAMs

**DOI:** 10.1002/advs.77308

**Published:** 2026-08-27

**Authors:** Yimin Fang, Jinghan Zhu, Qian Xiao, Qichun Wei, Xiujing He, Ping Li, Kai Jiang, Yue Liu, Yeting Hu, Weiwei Yu, Chuansheng Guo, Sheng Chen, Jian Zhao, Siqi Dai, Jingsun Wei, Hang Yang, Tongtong Bu, Kefeng Ding, Haiyan Chen

**Affiliations:** ^1^ Department of Colorectal Surgery and Oncology (Key Laboratory of Cancer Prevention and Intervention China National Ministry of Education) The Second Affiliated Hospital Zhejiang University School of Medicine Hangzhou Zhejiang China; ^2^ Center for Medical Research and Innovation in Digestive System Tumors Ministry of Education Hangzhou Zhejiang China; ^3^ Zhejiang Provincial Clinical Research Center for Cancer Hangzhou Zhejiang China; ^4^ Cancer Center of Zhejiang University Hangzhou Zhejiang China; ^5^ Department of Gynecology The Second Affiliated Hospital Zhejiang University School of Medicine Hangzhou Zhejiang China; ^6^ Department of Radiation Oncology (Key Laboratory of Cancer Prevention and Intervention, China National Ministry of Education) The Second Affiliated Hospital, Zhejiang University School of Medicine Hangzhou Zhejiang China; ^7^ Institute of Breast Health Medicine State Key Laboratory of Biotherapy West China Hospital Sichuan University and Collaborative Innovation Center Chengdu Sichuan China; ^8^ Department of Radiation Therapy Jinhua Municipal Central Hospital Jinhua Zhejiang China; ^9^ Center for Basic and Translational Research the Second Affiliated Hospital School of Medicine Zhejiang University Hangzhou Zhejiang China

**Keywords:** colorectal cancer, M2 polarization, radiotherapy resistance, small extracellular vesicles, tumor‐associated macrophages

## Abstract

**Background and Aims:**

Tumors actively remodel the tumor microenvironment (TME) to foster therapy resistance. However, the underlying mechanisms remain poorly defined. This study aims to characterize how colorectal cancer (CRC) cells drive TME remodeling to promote radioresistance.

**Methods:**

A radioresistant CRC cell line was established via serial in vivo irradiation. Single‐cell RNA sequencing characterized TME alterations. The role of tumor‐derived small extracellular vesicles (sEVs) in macrophage polarization was assessed. Proteomic analysis identified ANXA2 as a key sEVs cargo, and calcium‐dependent sEV secretion was investigated through SLC8A1 modulation. Clinical relevance analyses were further assessed.

**Results:**

The radioresistant model exhibited enhanced treatment resistance to irradiation and increased infiltration of M2‐like tumor‐associated macrophages (TAMs), while TAM depletion restored radiosensitivity. Tumor‐derived sEVs promoted M2 polarization by activating STAT6 phosphorylation, with ANXA2 as the key functional cargo. Further analysis revealed that SLC8A1 deficiency increased intracellular Ca^2^^+^ levels, boosting secretion of ANXA2^+^ sEVs and subsequent TAM M2 polarization. Clinically, low SLC8A1 expression correlated with higher M2 TAM infiltration, poorer therapeutic response, and worse survival outcomes in CRC patients.

**Conclusion:**

CRC cells with low SLC8A1 expression remodel the TME to foster radioresistance via calcium‐dependent secretion of ANXA2^+^ sEVs and subsequent M2 polarization of TAMs.

## Graphical abstract

SLC8A1 deficiency in colorectal cancer cells increases intracellular calcium signaling, promoting the secretion of ANXA2‐enriched small extracellular vesicles. These vesicles reprogram tumor‐associated macrophages towards an M2 phenotype, which in turn fosters a tumor microenvironment conducive to radioresistance and is associated with poor patient outcomes.

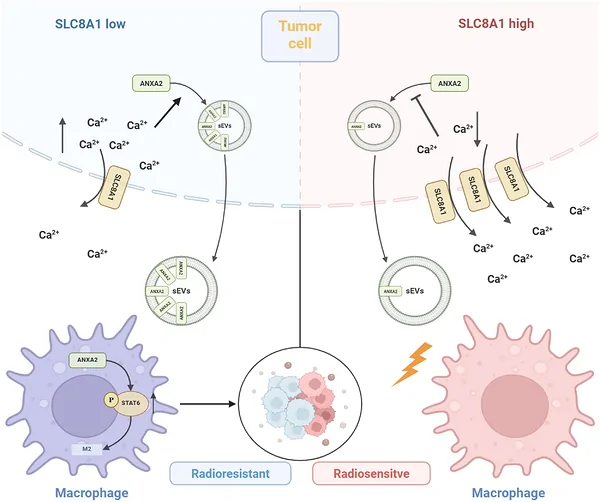

## 1 Introduction

Radiotherapy is essential for treating colorectal cancer (CRC), significantly improving locoregional control and survival outcomes [1]. This is particularly true for rectal cancer, where it not only enhances therapeutic efficacy but also facilitates organ preservation, specifically anal sphincter function [2, 3]. Nevertheless, considerable inter‐individual variability exists. While some patients (about 20%) achieve a complete response to radiotherapy‐based treatment, others exhibit resistance [4, 5]. Therefore, elucidating the molecular mechanisms underlying radioresistance in CRC represents a fundamental prerequisite for optimizing radiotherapeutic efficacy and improving clinical outcomes.

Tumor heterogeneity not only directly influences treatment response but also remodels the tumor microenvironment (TME) to promote progression and drive resistance [6, 7]. The TME is a complex network of non‐tumor cells and acellular components surrounding tumor cells. For instance, tumor‐associated macrophages (TAMs), the predominant TME component, secrete arginase‐1 and IL‐10 to inhibit T‐cell function [8, 9, 10, 11]. Regulatory T cells suppress cytotoxic immunity via TGF‐β signaling [12], and myeloid‐derived suppressor cells drive T‐cell exhaustion, to collectively establish a radio‐protective ecosystem within the TME that compromises treatment efficacy [8, 13, 14]. The dynamic crosstalk between tumor cells and the TME complicates anticancer therapy, making it critical to elucidate how cancer cells remodel the TME to promote radioresistance and to characterize the molecular features of radioresistant CRC cells. Tumor cells employ multiple mechanisms to remodel the immunosuppressive niche, including direct cellular engagement (e.g., PD‐L1/PD‐1 interactions) [15], paracrine cytokine signaling [16], and extracellular vesicle‐mediated reprogramming [17].

Small extracellular vesicles (sEVs), membrane‐bound particles with diameters <200 nm [18, 19], are recognized as critical mediators in tumor‐TME crosstalk [19, 20]. By transferring bioactive molecules to recipient cells within the TME [21], sEVs allow tumor cells to orchestrate immunosuppressive signaling, thereby drive tumor proliferation, migration, and resistance [22]. Our previous studies showed that plasma‐derived sEV protein‐associated genes can predict chemoradiotherapy response and prognosis in patients with locally advanced rectal cancer (LARC) [23], and demonstrated that ANGPTL1^+^ exosomes suppress vascular leakiness and liver metastasis in CRC [24]. Besides, accumulating evidence indicates that inhibiting the secretion of tumor‐derived sEVs suppresses tumor progression [25] and enhances therapeutic sensitivity [26]. While sEVs are established mediators of treatment resistance in cancers like glioblastoma—where specific cargo such as circATP8B4 is proven to transfer radioresistance via sEVs [27] —a critical and unresolved gap exists in CRC. It remains unknown whether CRC‐derived sEVs similarly drive resistance to radiotherapy, particularly through reprogramming the TME via mechanisms like TAMs polarization. This gap directly underscores the necessity of the present work, which aims to clarify whether CRC‐derived sEVs are involved in regulating radiotherapy resistance and, if so, how they exert this regulatory function.

This study aims to determine whether CRC cells actively promote radiotherapy resistance by orchestrating immunosuppressive TME remodeling through sEVs, dissecting the mechanisms underlying sEV‐mediated TME reprogramming, and identifying the key intracellular regulators that govern this resistance‐promoting cascade.

## 2 Materials and Methods

### 2.1 Cell Culture and Construction of Radioresistant Cells

Two radioresistant cell lines were generated using distinct induction strategies: MC38‐RR was established through serial in vivo irradiation (IR) and passaging to preserve influences from TME, while MC38‐RRvt was generated via repeated in vitro IR as previously described [28].

To generate the MC38‐RR line, MC38 cells were subcutaneously injected into C57BL/6 mice. Once tumors reached 100–150 mm^3^, they were locally irradiated with 10 Gy. When tumors grew to approximately 500 mm^3^, they were collected, cut into small fragments, and re‐transplanted into new mice. This in vivo selection process was repeated five times. After the final cycle, tumors were harvested and dissociated into primary cells, which were then cultured and stabilized for further use. Extensive frozen stocks were prepared from early passages immediately after stabilization, and all experiments were completed within 10 passages after thawing to minimize phenotype drift associated with long‑term culture.

Both radioresistant variants, along with parental MC38 cells (ATCC), were maintained in RPMI‐1640 supplemented with 10% FBS at 37°C under 5% CO_2_.

### 2.2 Western Blotting (WB)

WB was performed as previously described [24, 29]. Briefly, protein expression levels were analyzed by WB using primary antibodies against the indicated target proteins (Table S1).

### 2.3 Quantitative Real‐Time Polymerase Chain Reaction (qRT‐PCR)

Total RNAs were isolated from cells with RNA Isolation Kit (Vazyme, China), and were reverse transcribed to cDNA by HiScript II Reverse Transcriptase (Vazyme). Quantitative PCR was conducted using SYBR‐Green PCR kits (YEASEN, China) and a 7500 Fast Real‐Time PCR System (Life Technologies, China). All the primers used were listed in Table S2.

### 2.4 Radiotherapy

Cells or subcutaneous tumors were irradiated with an X‐ray beam from a linear accelerator (Siemens, Munich, Germany), giving the cells (6 Gy) or mice (8 Gy) a highly uniform and precise dose of radiation. The linear accelerator produces an X‐ray photon beam (6 MV) with a dose rate of 3 Gy min^−1^.

### 2.5 Subcutaneous Tumorigenesis Model

Female C57BL/6 mice (6‐8 weeks old, Zhejiang Laboratory Animal Center) were subcutaneously injected with 5×10^5^ CRC cells in 100 µL PBS. Tumor‐bearing mice were randomized for radiotherapy when volumes reached 100–150 mm^3^, with growth monitored every 2–3 d using caliper measurements (volume = ½×length×width^2^). For in vivo sEV education, mice received intratumoral injection of sEVs (10 µg/20 µL) every other day for a total of three doses, alternating with radiotherapy (8 Gy every other day) on the intervening days. Animal studies were approved by the Animal Care and Use Committee of the Second Affiliated Hospital Zhejiang University School of Medicine (Approval Number: 2022–222).

### 2.6 Single‐Cell RNA Sequencing (scRNA‐seq)

Resected MC38/MC38‐RR tumors (with/without radiotherapy) from five mice per group were pooled, PBS‐rinsed, and dissociated into single‐cell suspensions. After assessment of viability by Trypan Blue exclusion, cells were processed on the 10x Chromium platform for cDNA amplification and sequencing following manufacturer's protocols.

### 2.7 Isolation of Bone Marrow‐Derived Macrophages (BMDMs)

Following ethanol sterilization (75%, 3 min) of euthanized mice, tibiae and femurs were aseptically processed to extract bone marrow cells via RPMI‐1640 flushing, 70 µm filtrations, and centrifugation (300 × *g*, 5 min). After erythrocyte lysis (3 min, RT) and washing, cells were cultured in RPMI‐1640 containing 10 ng mL^−1^ M‐CSF (Novoprotein, China), with medium changes every 72 h, yielding adherent mature macrophages by day 7. For sEV education, BMDMs were treated with sEVs (10 µg mL^−1^) isolated from indicated groups for 24 h.

### 2.8 Immunohistochemistry (IHC)

Following standard paraffin processing (4% PFA fixation, xylene deparaffinization, ethanol rehydration) and antigen retrieval (LAB Solution, 5 min, 25°C), tissue sections (4 µm) were immunostained using the UltraSensitive SP IHC Kit (Maixin). Briefly, slides underwent sequential procedures including: 1) peroxidase blocking (10 min), 2) nonspecific binding reduction (goat serum), 3) primary antibody incubation (anti‐SLC8A1, 1:100, Abcam #ab2869; 4°C overnight), 4) DAB visualization, and 5) hematoxylin counterstaining.

### 2.9 Multiplex Immunohistochemical (mIHC)

Following standard IHC processing, tissue sections underwent iterative staining cycles: primary antibodies (F4/80, 1:200, CST, #70076; CD206, 1:200, CST, #24595; 4°C overnight) with HRP‐secondaries (50 min, RT) and TSA amplification (iF488, 10 min dark), alternating with microwave stripping (95°C, 10 min). Final processing included DAPI counterstaining (10 min), autofluorescence quenching, and spectral unmixing imaging for multiplex signal acquisition.

### 2.10 Differentiation of THP‐1 and Co‐Culture System

THP‐1 cells (ATCC) were seeded into 6‐well plates and differentiated into macrophages by stimulation with 100 ng mL^−1^ PMA (MCE) for 24 h. Subsequently, the medium was replaced with fresh PMA‐free medium, and the cells were cultured for an additional 24 h prior to use. The resulting THP‐1‐derived macrophages were then treated with sEVs (10 µg mL^−1^) from indicated groups for 24 h. For co‐culture experiments, sEV‐educated macrophages were seeded into the upper chamber of a transwell insert (Corning), which was placed into 12‐well plates containing pre‐seeded RKO cells in the lower chambers. The cells were co‐cultured for 24 h and then the entire co‐culture system was subjected to IR (6 Gy). The effect of macrophages on the radiosensitivity of RKO cells was assessed using CCK‐8 and colony formation assays.

### 2.11 Construction of Knockdown, Knockout, and Overexpression Cell Lines

Stable knockdown cell lines were generated by transducing cells with commercially prepared lentiviral vectors (GenePharma, China) targeting RAB27A, ANXA2, SLC8A1 or corresponding control sequences at a multiplicity of infection of 5–20 TU per cell, followed by puromycin selection (10 µg mL^−1^, 48–72 h post‐transduction). For transient knockdown in BMDMs, cells were transfected with STAT6‑specific siRNA or control siRNA (GenePharma, China) using Lipofectamine RNAiMAX (Invitrogen, USA) according to the manufacturer's instructions at a final concentration of 100 nm. For CRISPR‑Cas9‑mediated knockout, MC38‑RR cells were transfected with a single‑vector system expressing both sgRNA targeting Anxa2 and Cas9 (GenePharma, China) using Lipofectamine 3000 (Invitrogen, USA). At 48 h post‑transfection, cells were subjected to puromycin selection (2 µg mL^−1^) for 72 h, and single‑cell clones were isolated by limiting dilution and verified by WB. For rescue experiments, ANXA2‑knockout cells were reconstituted with wild‑type ANXA2 or the calcium‑binding‑deficient E96A mutant via lentiviral transduction, followed by puromycin selection (2 µg mL^−1^), and the resulting cell lines were used for subsequent functional analyses.

### 2.12 Extraction of sEVs

For basal sEV collection, tumor cells were cultured in sEV‐depleted medium for 72 h. For sEVs induced by IR, cells were exposed to 6 Gy radiation and subsequently cultured under identical conditions. In both cases, the supernatant was then processed through sequential centrifugation: 500 × *g* for 10 min to remove cells, 3000 × *g* for 20 min for apoptotic bodies, and 10 000 × *g* for 20 min for micro vesicles. sEVs were finally isolated by two rounds of ultracentrifugation (100 000 × *g*, 70 min each; Beckman Ti70 rotor).

### 2.13 sEV Labeling

The isolated sEVs were labeled with the PKH67 membrane dye (Sigma, Germany, PKH67GL). To remove unincorporated dye, the labeled sEVs underwent a third round of ultracentrifugation at 100 000 × *g* for 70 min. The labeled sEVs were then pelleted and resuspended in PBS.

### 2.14 Immunoelectron Microscopy (Immune‐EM)

MC38 cells treated with 500 nm calcimycin or vehicle for 3 h were fixed with 2% paraformaldehyde and 0.1% glutaraldehyde for 15 min at room temperature, permeabilized with 0.1% saponin for 40 min, and blocked with 0.1% BSA for 1 h. Samples were then incubated overnight at 4°C with anti‑ANXA2 primary antibody (Huabio, ET1704‑49, 1:100), followed by FluoroNanogold‑labeled anti‑rabbit secondary antibody for 1 h at room temperature. After post‑fixation with 2.5% glutaraldehyde and silver enhancement, ultrathin sections were prepared using an ultramicrotome following 48 h of polymerization at 60°C and examined by TEM (Tecnai G2 Spirit, 120 kV).

### 2.15 Flow Cytometry

Cell suspensions were washed (PBS, 350 × *g*, 5 min) and Fc‐blocked (BD, USA, #553142, 5 min) prior to surface staining with anti‐F4/80‐BV421 (biolegend, USA, #123137, 20 min, RT, dark). After fixation and permeabilization (biolegend, USA, #421002, 20 min, RT), intracellular staining was performed with anti‐CD206‐AF647 antibody (biolegend, USA, #141712, 20 min, RT, dark). Cells were washed intermittently with PBS (350 × *g*, 5 min) throughout the staining procedure. Prepared samples were analyzed by flow cytometry (BD FACS Canto II).

### 2.16 LC‐MS/MS‐Based Proteomic Analysis

sEV proteins were processed through reduction (10 mm DTT, 56°C, 30 min), alkylation (55 mm IAA, dark, RT, 15 min), and tryptic digestion (1:50 enzyme: substrate, 37°C, overnight) before LC‐MS/MS analysis (Guangke Ande Biotechnology). Peptides were acidified (0.1% TFA), desalted (C18 StageTips), and separated on a self‐packed C18 column (20 cm × 75 µm; 1.9 µm) using a 120‐min gradient (Easy‐nLC 1200 UHPLC/Q Exactive HF‐X) in DDA mode.

### 2.17 RNA Sequencing

RNA sequencing was performed by Gene Denovo (Guangzhou, China) on MC38 and MC38‐RR cells. Differentially expressed genes (DEGs) were identified using the DEGseq R package with thresholds of |log2FC|≥1 and FDR≤0.01. Subsequently, KEGG pathway enrichment analysis was conducted to identify key pathways associated with radiation response.

### 2.18 Regulation and Measurement of Intracellular Calcium Ion Concentration

To modulate intracellular Ca^2^^+^ levels, MC38 cells were treated with 500 nm calcimycin (24 h) to increase intracellular Ca^2^^+^ concentration ([Ca^2^^+^]_i_), whereas MC38‐RR cells were treated with 10 µm BAPTA‐AM for 24 h to chelate intracellular Ca^2^. For [Ca^2^^+^]_i_ measurement, cells were loaded with 0.5 µm Fluo‐4 AM (20 min, RT), washed, and incubated (20–30 min) for complete de‐esterification. Fluorescence intensity reflecting [Ca^2^^+^]_i_ levels were analyzed by flow cytometry (BD FACS Canto II) and fluorescence microscopy (Nikon Eclipse Ti; Ex/Em: 494/506 nm).

### 2.19 Clinical Sample Collection and Analysis

This study used clinical samples from treatment‐naïve LARC patients (Jinhua Municipal Central Hospital, 2013–2016) who completed neoadjuvant chemoradiotherapy (nCRT) and total mesorectal excision. Patients were stratified according to pathological response status into pathological complete response (pCR) and non‐pCR groups, with comparable baseline characteristics between groups (Table S3). The density of M2‐TAMs (CD68^+^CD163^+^) was quantified in five randomly selected 400× fields (cells mm^−^^2^).The optimal cutoff value for M2‐TAM infiltration was determined using the surv_cutpoint function to classify patients into high‐ and low‐infiltration groups. The same analytical workflow was applied to evaluate M2‐TAM infiltration and SLC8A1 expression by IHC. Survival analyses were performed using Kaplan–Meier methods with the survminer and ggplot2 packages. Approval was granted by the Ethics Committee of Jinhua Municipal Central Hospital (Approval Number: 2018R‐154).

### 2.20 Public Database Collection and Analysis

Gene expression and clinical data were integrated from two independent cohorts: the Memorial Sloan Kettering Cancer Center (MSK) cohort of LARC patients (https://www.cbioportal.org/study/summary?id = msk_impact_2017)and the Cancer Genome Atlas (TCGA) colorectal adenocarcinoma cohort (https://portal.gdc.cancer.gov/)). The MSK cohort was initially composed of 100 LARC patients. Patients were included based on the following criteria: 1) receipt of nCRT‐based treatment; and 2) a pre‐treatment AJCC stage of II‐III (LARC). After applying these criteria and excluding 5 patients due to missing survival data, a final cohort of 84 patients was retained for analysis. GEPIA analysis of TCGA data (305 colorectal tumors vs 667 normal tissues) revealed differential SLC8A1 expression. In the MSK cohort, the prognostic relevance of M2 macrophage infiltration was assessed by stratifying patients into high‐ and low‐infiltration groups with comparable baseline characteristics (Table S4). Optimal cutoff values for SLC8A1 expression and M2 macrophage infiltration were determined using maximally selected rank statistics (surv_cutpoint), followed by Kaplan–Meier survival analysis (survfit) and visualization using the ggplot2 and ggpubr packages.

### 2.21 Statistical Analysis

All experimental data processing and graphical generation were performed using GraphPad Prism 9.0, with statistical comparisons implemented as follows: unpaired t‐tests for independent group comparisons, paired t‐tests for matched samples, one‐way ANOVA with Tukey's post‐hoc analysis for multi‐group comparisons, log‐rank tests for survival curve analyses, and Pearson's correlation for association studies, where statistical significance was defined as *P* < 0.05.

## 3 Results

### 3.1 M2‐Like TAMs Promote Radioresistance and Contribute to Poor Survival in CRC

To overcome the limitations of conventional in vitro radioresistance induction methods that neglect TME contributions, we established a novel MC38‐RR cell line through serial in vivo IR and passaging in mice (Figure 1A). Compared to parental MC38 and in vitro‐derived radioresistant MC38‐RRvt cells, MC38‐RR tumors exhibited significantly greater volume and weight in mice after radiotherapy (Figure 1B–D). Concurrently, following IR, MC38‐RR cells exhibited significantly higher cell viability and reduced levels of lipid peroxidation compared to parental MC38 cells, although no significant difference in the apoptosis rate was observed (Figure S1A–C).

**FIGURE 1:**
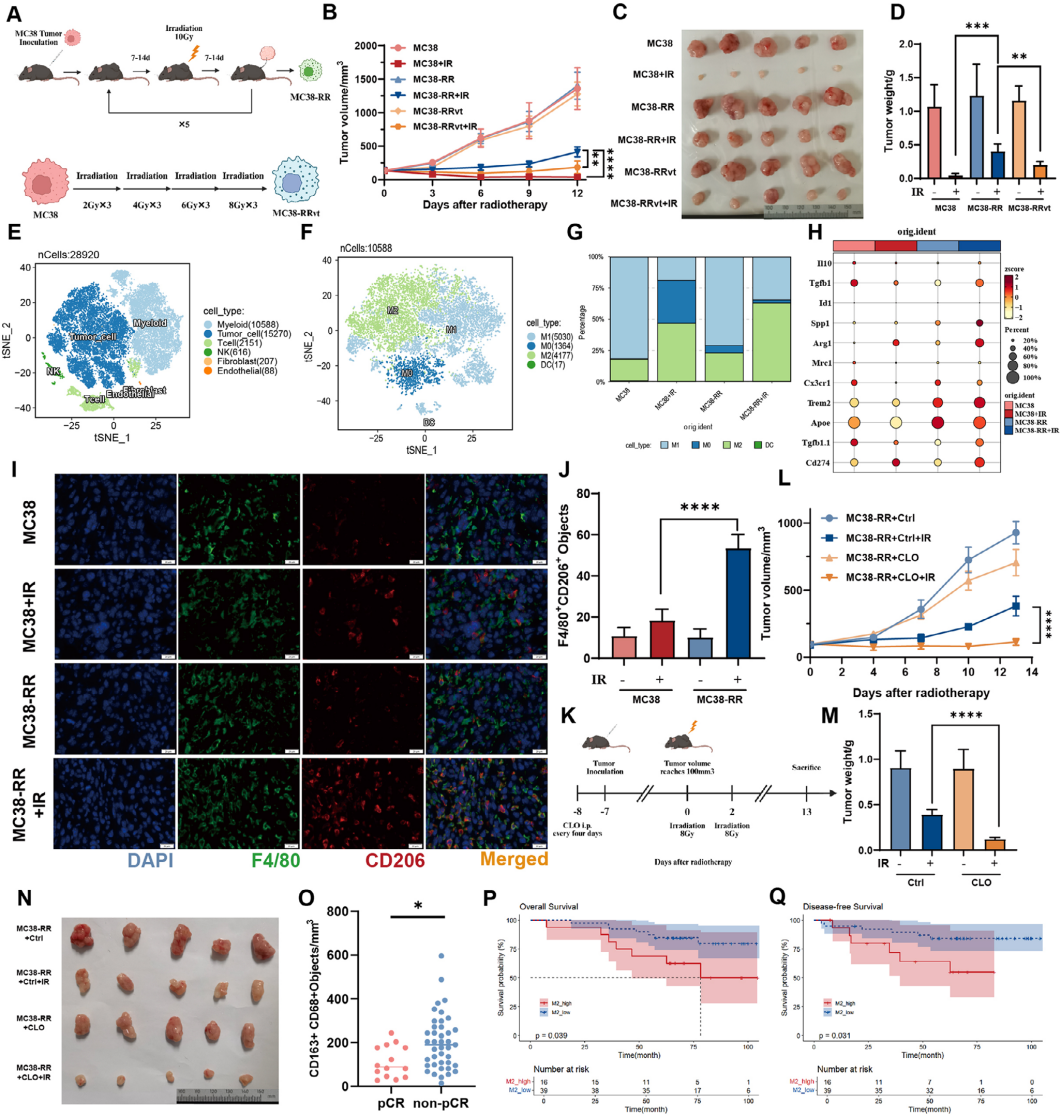
M2‐Like TAMs promote radioresistance and contribute to poor survival in CRC. (A) Schematic of radioresistant cell line establishment (in vivo MC38‐RR; in vitro MC38‐RRvt). (B–D) Tumor growth kinetics post‐radiotherapy (8 Gy × 2 at 100 mm^3^): Growth curves (B), tumor sizes (C), and masses (D) of MC38, MC38‐RR, and MC38‐RRvt subcutaneous tumors (*n* = 5). (E–H) scRNA‐seq analysis of tumor immune microenvironment: (E) tSNE plot clustering. (F) Monocyte/macrophage subclustering. (G) Cellular proportion per subpopulation. (H) Immunosuppressive gene expression in TAMs. (I, J) mIHC of irradiated tumors: (I) Representative images (DAPI: nuclei; F4/80: green; CD206: red). (J) Quantified F4/80^+^CD206^+^ cells/HPF (*n* = 6). (K–N) Macrophage depletion with CLO (5 mg mL^−1^, 200 µL per mouse q4d): (K) Treatment timeline. (L–N) Tumor growth curves (L), masses (M), and sizes (N) in irradiated MC38‐RR tumors (*n* = 5). (O–Q) Association between M2‐TAMs infiltration and patient outcomes: (O) CD68^+^CD163^+^ cell density in post‐neoadjuvant pCR versus non‐pCR tissues. (P, Q) Survival analysis in LARC patients: OS (P) and DFS (Q) stratified by M2‐TAMs infiltration. Statistical analyses: One‐way ANOVA (multiple groups), unpaired t‐test (two groups), log‐rank test (survival). Significance: *p < 0.05, **p < 0.01, ***p < 0.001, ****p < 0.0001.

To characterize the TME features associated with acquired radioresistance, we performed scRNA‐seq of parental MC38 and MC38‐RR tumors (Figure 1E and Figure S1D). Immune profiling revealed that monocyte‐derived macrophages and T cells as predominant immune components (Figure S1E, F). Given their prominence, deeper analysis of the monocyte/macrophage population revealed increased M2 macrophage infiltration in irradiated MC38‐RR tumors (Figure 1F,G), accompanied by significant upregulation of immunosuppression‐associated genes (Id1, Il10, Spp1, Arg1, Mrc1, Cx3cr1, Trem2, Apoe, Tgf‐β1, Cd274) in these cells post‐IR (Figure 1H). While T‐cell analysis showed no significant difference in CD8^+^T cell distribution, Tregs exhibited increased infiltration in MC38‐RR tumors (Figure S1G,H). These findings suggest increased M2 TAMs and Treg infiltration is associated with CRC radioresistance. Given the more prominent expansion and immunosuppressive phenotype of M2‐like TAMs, subsequent studies focused on their contribution to CRC radioresistance. mIHC confirmed significantly higher M2 TAMs (F4/80^+^CD206^+^) infiltration in irradiated MC38‐RR tumors compared to all other groups (Figure 1I,J). To determine whether TAMs directly contribute to radioresistance, we depleted macrophages in MC38‐RR tumors using clodronate liposomes (CLO) (Figure S1I) prior to radiotherapy (Figure 1K). Compared to control liposomes, macrophage depletion significantly reduced tumor volume and weight post‐IR (Figure 1L–N), indicating restored radiosensitivity. Collectively, these findings demonstrate that radioresistant CRC tumors acquire an immunosuppressive TME characterized by increased M2 TAM infiltration, which actively promotes resistance to radiotherapy.

We next investigated the clinical relevance of M2 TAM (CD68^+^CD163^+^ cells) infiltration in CRC patients undergoing radiotherapy. Analysis of paired pre‐ and post‐ nCRT biopsies (Figure S1J) revealed that M2 TAM density remained unchanged in patients achieving pathological complete response (pCR) (Figure S1K), whereas it was significantly increased after treatment in patients without pCR (non‐pCR group) (Figure S1L). Moreover, post‐nCRT tumors from non‐pCR patients exhibited significantly higher M2 TAM infiltration compared with pCR tumors (Figure 1O). Critically, patients with high post‐treatment M2 infiltration exhibited significantly worse overall survival (OS) and disease‐free survival (DFS) than those with low infiltration (Figure 1P,Q), consistent with findings from the MSK public database analysis (Figure S1M,N). These results demonstrate that increased infiltration of M2 TAMs is associated with impaired tumor regression and worse prognosis following radiotherapy in CRC.

### 3.2 Tumor‐Derived sEVs Reprogram TAMs toward an M2 Phenotype to Promote CRC Radioresistance

Given that tumor cells utilize secreted sEVs for immune regulation [30], we elucidated tumor‐mediated M2 polarization by educating BMDMs with conditioned medium (CM), sEVs, or sEV‐depleted CM from MC38/MC38‐RR cells with or without IR. The characteristics of sEVs were accessed by WB, transmission electron microscopy, and dynamic light scattering (Figure S2A–C). CM and sEVs from irradiated MC38‐RR cells significantly promoted M2 polarization, while sEV‐depleted CM failed to induce this phenotype (Figure S2D–I). Subsequently, sEVs from irradiated MC38/MC38‐RR cells were used to educate MC38 subcutaneous tumors (Figure 2A). Notably, tumors educated with sEVs from irradiated MC38‐RR cells exhibited significantly greater radioresistance compared with those receiving sEVs from irradiated MC38 cells (Figure 2B–D). Besides, Tumors educated with MC38‐RR‐IR‐derived sEVs displayed significantly increased infiltration of M2 TAMs (Figure 2E,F). To determine whether macrophages are required for sEV‐mediated radioresistance, macrophages were depleted before sEV administration (Figure 2G). Macrophage‐depleted tumors exhibited restored radiosensitivity (Figure 2H–J), demonstrating that sEV‐conferred radioresistance is macrophage‐dependent. To provide a physical basis for this functional delivery, PKH67‐labeled sEVs were efficiently internalized by BMDMs in vitro and by TAMs in vivo (Figure S3J,K).

**FIGURE 2:**
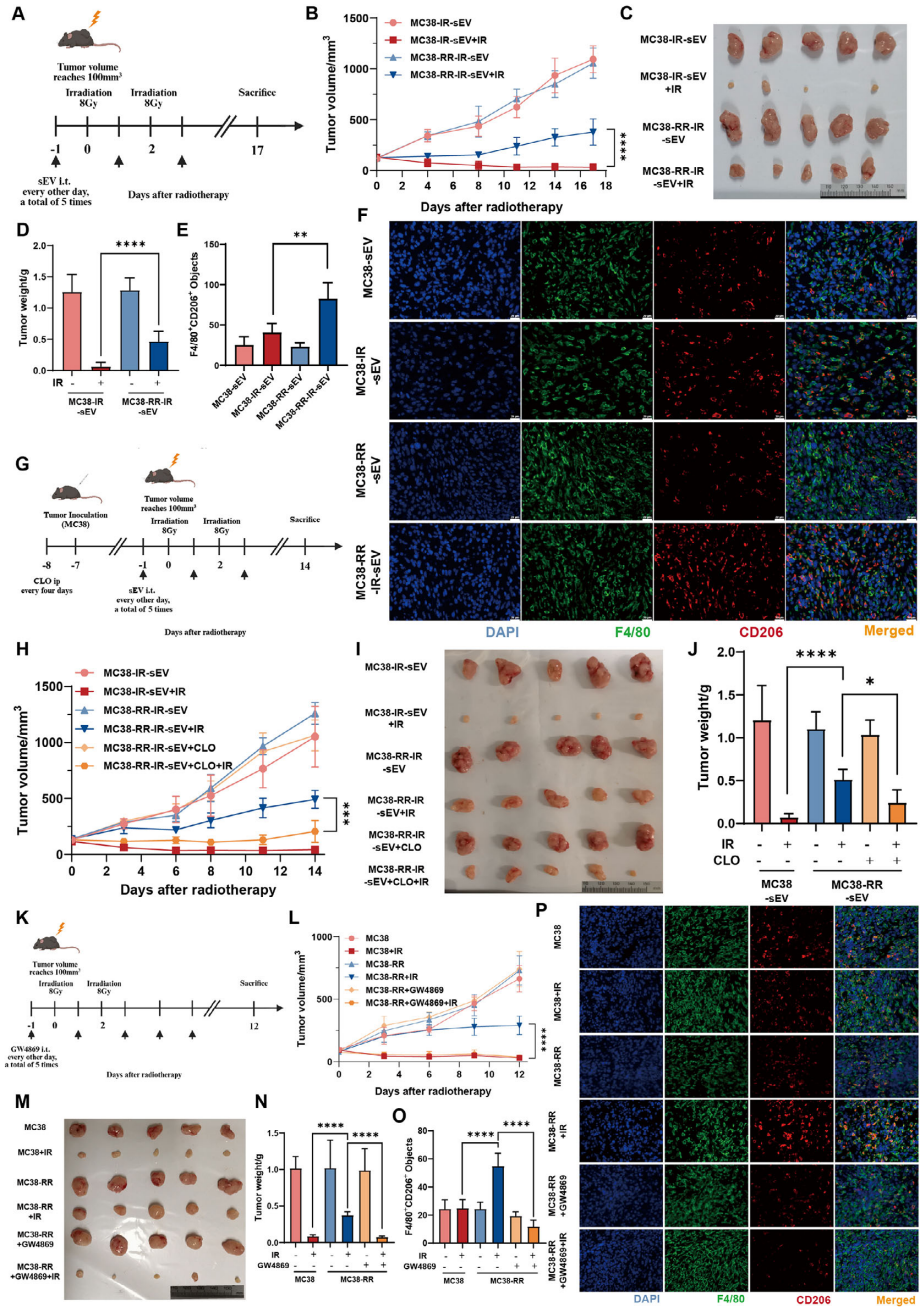
Tumor‐derived sEVs reprogram TAMs toward an M2 phenotype to promote CRC radioresistance. (A) Schematic diagram of the in vivo sEVs‐education experiment: MC38 tumors (100 mm^3^) received intratumoral injection with sEVs from irradiated MC38/MC38‐RR cells (10 µg/20 µL qod×3) + radiotherapy (8 Gy×2). (B–D) Tumor growth kinetics: growth curves (B), tumor sizes (C), and masses (D) (*n* = 5). (E, F) Tumor immunofluorescence 48 h post‐sEVs education (10 µg/20 µL, it): (E) Representative images (DAPI: blue; F4/80: green; CD206: red). (F) F4/80^+^CD206^+^ cells/HPF quantification (*n* = 6). (G) Macrophage depletion + sEVs education schema: CLO (5 mg mL, 200 µL/mouse q4d, ip) pre‐inoculation + sEVs/radiotherapy at 100 mm^3^ (sEVs‐education as A). (H–J) Tumor growth: growth curves (H), tumor sizes (I), masses (J) (*n* = 5). (K) Schematic diagram of in vivo GW4869‐mediated sEVs secretion blockade: MC38‐RR tumors (100 mm^3^) received GW4869 (10 mg kg^−1^ qod×5, it) + radiotherapy (8 Gy×2). (L–N) Tumor growth: curves (L), sizes (M), masses (N) (*n* = 5). (O, P) Concomitant M2‐TAMs analysis: (O) F4/80^+^CD206^+^ cells/HPF quantification. (P) Representative images (standard fluorescence) (*n* = 6). Statistics: One‐way ANOVA (multiple groups), unpaired t‐test (two groups). Significance: *p < 0.05, **p < 0.01, ***p < 0.001, ****p < 0.0001.

To further establish the specific contribution of CRC‐derived sEVs, we pharmacologically and genetically disrupted sEV biogenesis and secretion using the neutral sphingomyelinase inhibitor GW4869 and Rab27a knockdown in MC38‐RR cells, respectively. Both approaches significantly attenuated the enhanced M2 polarization of BMDMs induced by co‐culture with irradiated MC38‐RR cells (Figure S3A–K). In vivo, administration of GW4869 during radiotherapy (Figure 2K) not only increased tumor radiosensitivity (Figure 2L,N), but also decreased M2 TAM (F4/80^+^ CD206^+^) infiltration in MC38‐RR tumors (Figure 2O,P). Likewise, Rab27a knockdown in tumors also enhanced radiosensitivity (Figure S3L–N). Collectively, these findings demonstrate that CRC‐derived sEVs function as critical mediators of macrophage reprogramming, driving M2‐like TAM polarization and establishing an immunosuppressive microenvironment that promotes tumor radioresistance.

### 3.3 ANXA2^+^SEVs Drive TAMs M2 Polarization via STAT6 Phosphorylation

Building on the findings that tumor‐derived sEVs drive M2 polarization and radioresistance, we sought to identify the key functional components responsible for these effects through proteomic profiling. Comparative analysis identified 60 and 72 upregulated proteins in sEVs derived from (i) MC38‐RR cells with or without IR and (ii) MC38‐RR cells compared with parental MC38 cells, respectively (Figure S4A,B). Intersection of these datasets revealed 18 commonly upregulated proteins (Table S5). Among these candidates, ANXA2, ERO1A and VCP exhibited consistently high fold changes in both comparisons. Subsequent WB validation showed that although both ANXA2 and VCP followed trends consistent with the proteomics data, but the difference was substantially more pronounced for ANXA2 than for VCP (Figure S4C), highlighting ANXA2 as a potential functional mediator.

To determine the biological role of ANXA2, we generated Anxa2‐knockdown cells (MC38‐RR‐shAnxa2, Figure S4D), which produced sEVs with markedly reduced ANXA2 abundance (Figure S4E). Notably, Anxa2 knockdown in tumor cells significantly attenuated their ability to induce M2 polarization in BMDMs (Figure 3A–E). Concomitantly, it enhanced tumor radiosensitivity in vivo, whereas this effect was conversely rescued by supplementation with sEVs derived from control cells (Figure 3F–H). Furthermore, a protease protection assay demonstrated that ANXA2 was resistant to proteinase K digestion in intact sEVs but became susceptible after Triton X‐100‐mediated membrane permeabilization (Figure S4F), indicating that ANXA2 is enclosed within sEVs rather than exposed on their surface. Thus, ANXA2 is a critical functional component of CRC‐derived sEVs that promotes M2 polarization and confers radioresistance.

**FIGURE 3:**
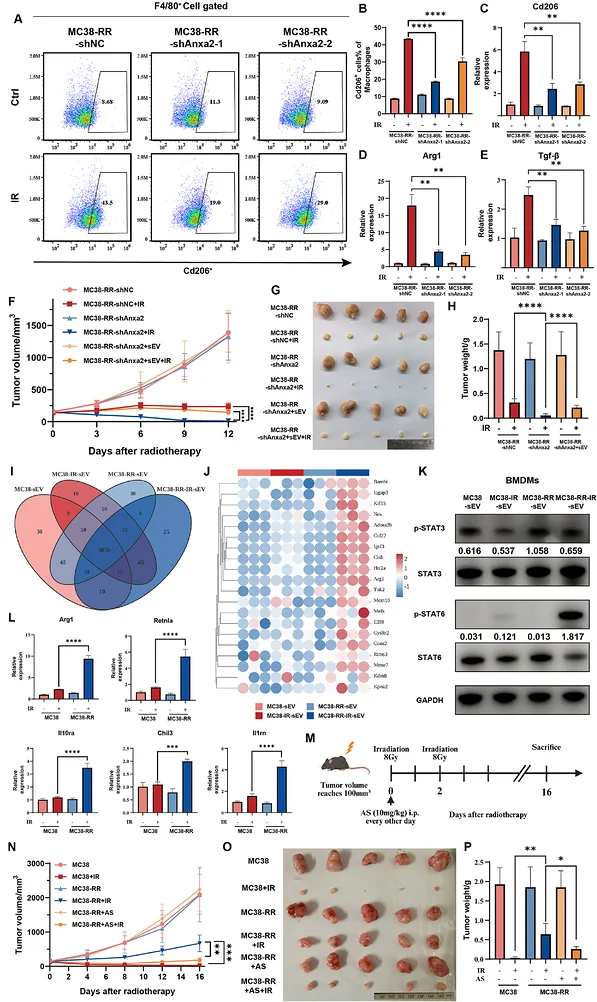
ANXA2^+^sEVs promote M2 polarization via STAT6 phosphorylation. (A–E) In vitro M2 polarization analysis: BMDMs educated for 24 h with sEVs from irradiated (6 Gy) or non‐irradiated MC38‐RR‐shNC/shAnxa2 cells. (A, B) Flow cytometry of M2 polarization. (C–E) qPCR of Cd206, Arg1, Tgf‐β mRNA (*n* = 3). (F–H) In vivo tumor radiosensitivity analysis. Subcutaneous tumors were established from MC38‐RR‐shNC, MC38‐RR‐shAnxa2, or MC38‐RR‐shAnxa2 cells replenished with sEVs from MC38‐RR‐shNC cells, and each group was then treated with or without radiotherapy (8 Gy×2 at tumor volume ≥100 mm^3^): Tumor growth curves (F), endpoint sizes (G), and masses (H) (*n* = 5 per group). (I–L) Transcriptional regulation mechanism: (I) Venn diagram of DEGs in sEVs‐educated BMDMs, (J) Heatmap of 25 signature DEGs (MC38‐RR‐IR‐sEV‐educated BMDMs), (K) WB of STAT3/STAT6 in sEVs‐treated BMDMs (24 h), (L) qPCR validation of Il1rn, Retnla, Chil3, Arg1, Il10ra. (M‐P) STAT6 inhibition in vivo: (M) Schema: AS (10 mg kg^−1^ q3d, ip) + radiotherapy (8 Gy×2 at 100 mm^3^), (N–P) tumor growth curves (N), sizes (O), masses (P) (*n* = 5). Statistics: One‐way ANOVA (multiple groups), unpaired t‐test (two groups). Significance: *p < 0.05, **p < 0.01, ***p < 0.001, ****p < 0.0001.

We performed transcriptome sequencing to elucidate how ANXA2‐enriched sEVs (ANXA2^+^sEVs) drive M2 polarization. BMDMs treated with MC38‐RR‐IR sEVs exhibited 25 DEGs (Figure 3I), which clustered hierarchically (Figure 3J) and were enriched in the JAK‐STAT pathway by KEGG analysis (Figure S4G). Given the critical roles of STAT3 and STAT6 signaling in M2 polarization [31], we assessed their activation status. MC38‐RR‐IR sEV treatment resulted in a marked increase in STAT6 phosphorylated (p‐STAT6), but p‐STAT3 showed only minor changes (Figure 3K). Consistently, transcriptomic data showed upregulation of STAT6 target genes (Figure S4H), which was validated by qPCR, including Il1rn, Retnla, Chil3, Arg1, and Il10ra (Figure 3L).

Pharmacological inhibition of STAT6 phosphorylation using AS1517499 (AS) markedly attenuated sEV‐induced M2 polarization in vitro (Figure S4I–M) and enhanced radiosensitivity in vivo (Figure 3M–P). To further confirm the requirement of STAT6 signaling, we knocked down STAT6 in BMDMs (Figure S4N) and then educated these cells with MC38‑RR‑IR‑sEVs. STAT6 depletion significantly impaired sEV‑induced M2 polarization (Figure S4O–S), confirming STAT6 as the critical downstream effector of ANXA2^+^sEV‐mediated M2 polarization of TAMs.

Together, these findings demonstrate that ANXA2^+^sEVs promote TAM M2 polarization and tumor radioresistance by activating STAT6 signaling, establishing an ANXA2^+^sEV‐STAT6 axis as a key mechanism underlying tumor immune adaptation after radiotherapy.

### 3.4 Calcium‐Dependent Sorting of ANXA2 into sEVs Promotes M2 Polarization of TAMs

ANXA2 is a calcium‐dependent phospholipid‐binding protein that undergoes calcium‐triggered translocation to the plasma membrane, a process implicated in its selective incorporation into sEVs [32]. To explore the mechanism underlying the enhanced enrichment of ANXA2 in sEVs derived from radioresistant tumor cells, we performed KEGG pathway analysis on DEGs between MC38 and MC38‐RR cells. Notably, Ca^2^^+^‐related signaling pathways was significantly enriched in MC38‐RR cells (Figure 4A), suggesting that altered calcium homeostasis may contribute to ANXA2 loading into sEVs. Consistently, although IR increased intracellular Ca^2^^+^ levels ([Ca^2^^+^]_i_) in both cell lines, MC38‐RR cells exhibited a substantially greater elevation compared with parental MC38 cells (Figure 4B,C). We therefore directly tested whether modulating [Ca^2^^+^]_i_ could regulate ANXA2 loading into sEVs. Treatment of MC38 cells with the calcium ionophore calcimycin (Cal) significantly increased [Ca^2^^+^]_i_ (Figure S5A–C), resulting in elevated ANXA2 levels in secreted sEVs without altering total sEV secretion (Figure 4D and Figure S5D). Immune‐EM further revealed that calcium elevation promoted ANXA2 redistribution from the cytoplasm to the plasma membrane (Figure 4E), providing morphological evidence supporting calcium‐dependent membrane recruitment of ANXA2 during sEV biogenesis. Critically, sEVs from these Cal‐treated cells enhanced M2 polarization of BMDMs in vitro (Figure 4F–J) and conferred tumor radioresistance in vivo (Figure 4K–M). Conversely, chelating intracellular Ca^2^^+^ with BAPTA‐AM (BA) during IR in MC38‐RR cells (Figure S5E) significantly reduced ANXA2 levels in their sEVs (Figure S5F), and these BA‐treated sEVs showed a diminished capacity to induce M2 polarization (Figure 4N–R). To further determine whether the calcium‑binding capacity of ANXA2 is required for its loading into sEVs and subsequent function, we generated MC38‑RR‑ANXA2KO cells and reconstituted them with either wild‑type ANXA2 or the calcium‑binding‑deficient E96A mutant [33] (Figure S5G). Following irradiation, we measured [Ca^2^^+^]_i_ in these cells and found no significant differences among the groups (Figure S5H). sEVs from ANXA2KO cells exhibited markedly reduced ability to induce M2 polarization in BMDMs. Reconstitution with wild‑type ANXA2 restored this capacity, whereas the E96A mutant only partially rescued the effect (Figure S5I–M), indicating that the calcium‑binding capacity of ANXA2 is indispensable for its pro‑M2 polarization function.

**FIGURE 4:**
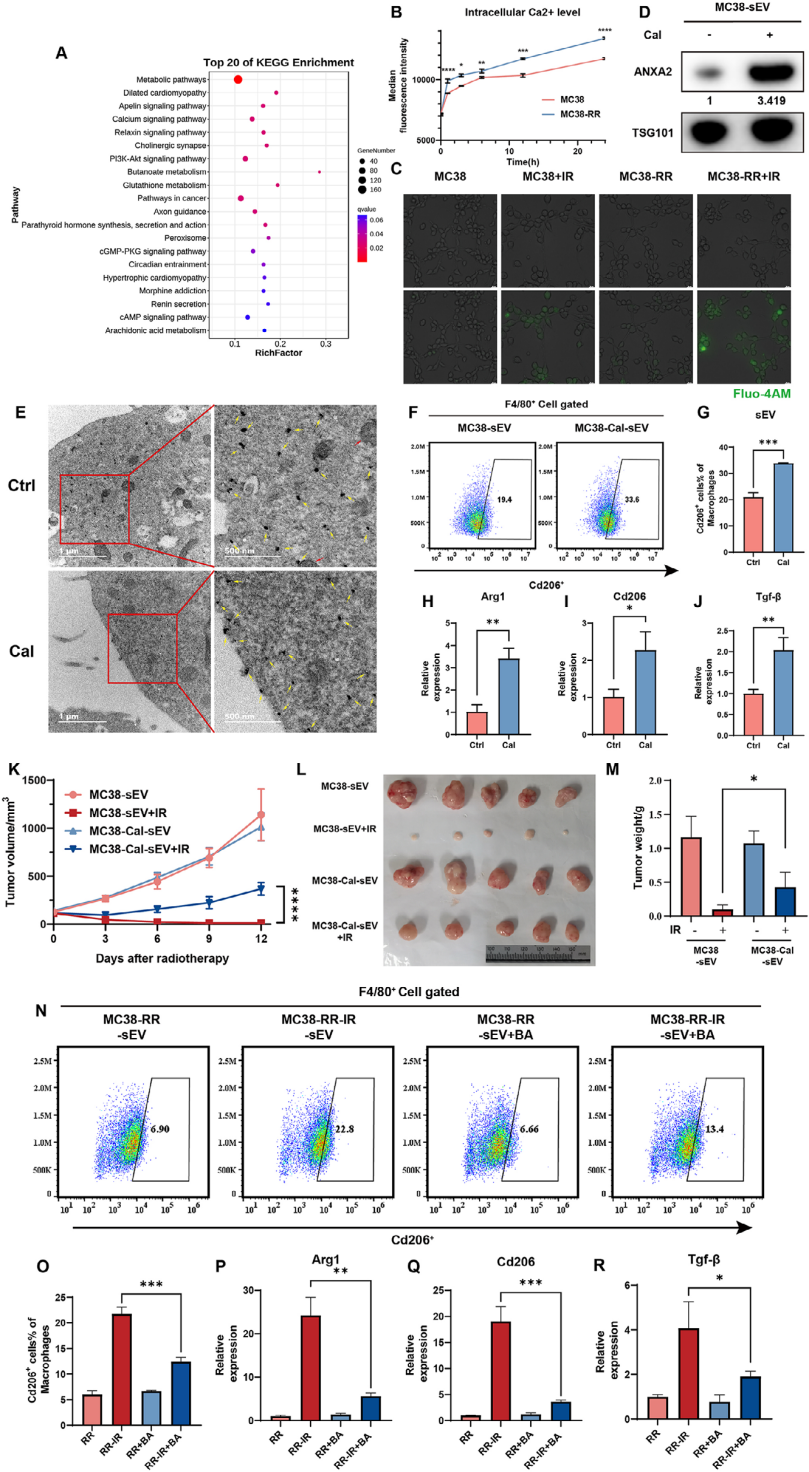
Calcium‐dependent loading of ANXA2 into sEVs drives M2 polarization. (A) KEGG pathway enrichment of DEGs (MC38‐RR vs MC38). (B, C) Radiation‐induced intracellular Ca^2^^+^ dynamics: (B) Flow cytometry quantification (Fluo‐4AM) at 0–24 h post‐6 Gy IR, (C) Representative fluorescence microscopy (24 h post‐IR) (*n* = 3). (D) ANXA2 elevation in sEVs from Cal‐treated (500 nm, 24 h) vs untreated MC38 cells. (E) Immune‐EM images showing the subcellular localization of ANXA2 (gold particles) in MC38 cells treated with or without Cal (500 nm, 3 h). Yellow arrows: Gold particles (ANXA2); Red arrows: mitochondria. (F‐J) Cal‐sEV effects on BMDMs (24 h education): (F‐G) Flow cytometry of M2 polarization, (H–J) qPCR of Cd206, Arg1, Tgf‐β (*n* = 3). (K–M) In vivo Cal‐sEV intervention: Tumors (100 mm^3^) received intratumoral injection with Cal treated/untreated sEVs (10 µg/20 µL qod×3) + radiotherapy (8 Gy×2). Growth curves (K), endpoint sizes (L), masses (M) (*n* = 5). (N–R) Calcium chelation effects: (N–O) Flow cytometry of M2 polarization in BMDMs educated with sEVs from MC38‐RR cells ± IR (6 Gy) ± BA (10 µm, 24 h), (P–R) Corresponding qPCR of Cd206, Arg1, Tgf‐β (*n* = 3). Statistics: Unpaired t‐test. Significance: *p < 0.05, **p < 0.01, ***p < 0.001, ****p < 0.0001.

To validate the causal link between of increased [Ca^2^^+^]_i_ and ANXA2^+^sEVs secretion, we extended these findings to the human CRC cell line RKO. Consistent with the results in murine cells, Cal treatment significantly increased ANXA2 enrichment in RKO‐derived sEVs (Figure S5N). These ANXA2^+^sEVs enhanced M2 polarization of THP‐1‐derived macrophages (Figure S5O,P). Crucially, when co‐cultured with RKO cells, macrophages educated by sEVs derived from Cal‐treated RKO cells significantly reduced the radiosensitivity of tumor cells (Figure S5Q–S).

Collectively, these data demonstrate that radiotherapy‐induced intracellular Ca^2^^+^ surge promotes ANXA2 recruitment into tumor‐derived sEVs, thereby driving M2 polarization of TAMs and fostering therapeutic resistance. This calcium‐dependent ANXA2^+^sEV axis represents a conserved mechanism underlying immune‐mediated radioresistance across murine and human CRC models.

### 3.5 SLC8A1 Deficiency Drives Radioresistance through Calcium‐Dependent ANXA2^+^sEVs Secretion and M2 TAM Polarization

To explore the mechanism underlying elevated [Ca^2^^+^]_i_ in irradiated MC38‑RR cells, calcium signaling‑related DEGs were analyzed and 33 dysregulated genes (Table S6) were identified between MC38‑RR and parental MC38 cells, among which SLC8A1 exhibited the most pronounced downregulation (Figure 5A). The reduced expression of SLC8A1 at the protein level was further confirmed by WB (Figure 5B). Other calcium flux‐regulating genes, Cacna1h, Itpr2 and Cacna1a did not show consistent expression patterns with the RNA‐seq data, and the downregulation of Atp2b4 was less pronounced than that of SLC8A1 (Figure S6A–D). Collectively, these findings identified SLC8A1 as the most significantly altered calcium‐regulatory gene in irradiated MC38‐RR cells, prompting us to further investigate its functional role in calcium homeostasis and radioresistance. Using Slc8a1‐knockdown cells (MC38‐shSlc8a1; Figure S5E), we demonstrated that SLC8A1 depletion resulted in increased post‐irradiation [Ca^2^^+^]_i_ (Figure S5F) and elevated ANXA2 enrichment in secreted sEVs (Figure 5D). These sEVs promoted M2 polarization of co‐cultured BMDMs (Figure 5E–I). In vivo, MC38‐shSlc8a1 subcutaneous tumors exhibited enhanced radioresistance and, consistent with the in vitro findings, showed significantly increased infiltration of M2 TAMs (F4/80^+^CD206^+^ cells) as revealed by mIHC (Figure S5G,H).

**FIGURE 5:**
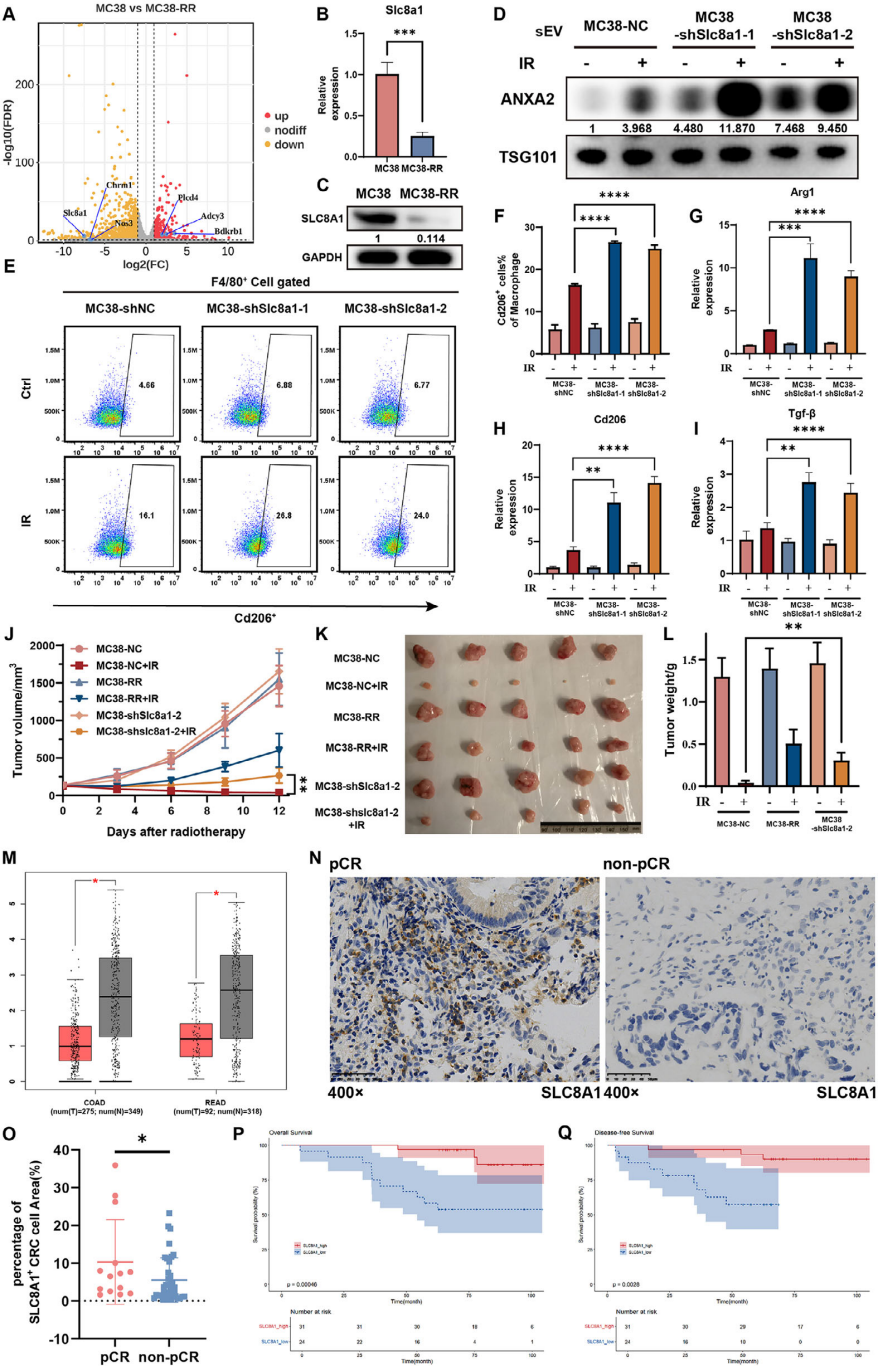
SLC8A1 deficiency drives radioresistance through calcium‐dependent ANXA2^+^sEVs secretion and M2 TAM polarization. (A) Volcano plot of DEGs (MC38 vs MC38‐RR) highlighting Ca^2^^+^ signaling genes. (B, C) Validation of reduced Slc8a1 mRNA (qPCR) and protein (WB) (*n* = 3). (D) ANXA2 elevation in sEVs from Slc8a1‐knockdown cells. (E‐I) Enhanced M2 polarization by MC38‐shSlc8a1‐IR‐sEVs (BMDMs educated 24 h with sEVs from irradiated (6 Gy) or non‐irradiated MC38‐NC, MC38‐shSlc8a1‐1, and MC38‐shSlc8a1‐2 cells). (E, F) Flow cytometry of M2 markers. (G–I) qPCR of Cd206, Arg1, Tgf‐β (*n* = 3). (J–L) In vivo radioresistance: MC38‐shSlc8a1‐2 vs MC38‐NC tumors receiving radiotherapy (8 Gy×2 at 100 mm^3^): Growth curves (J), endpoint sizes (K), masses (L) (*n* = 5). (M–Q) Clinical relevance: (M) Reduced SLC8A1 in COAD/READ tumors vs normal (TCGA). (N, O) Lower SLC8A1 in non‐pCR vs pCR patients (IHC). (P, Q) Poorer OS/DFS with low SLC8A1 (Kaplan‐Meier). Statistics: One‐way ANOVA (multiple groups), unpaired t‐test (two groups), log‐rank test (survival). Significance: *p < 0.05, **p < 0.01, ***p < 0.001, ****p < 0.0001.

Similar to murine cells, knockdown of SLC8A1 in RKO cells increased ANXA2 abundance in secreted sEVs (Figure S6I,J). These ANXA2‐enriched sEVs enhanced M2 polarization of THP‐1‐derived macrophages (Figure S6K,L). Furthermore, in a co‐culture system, macrophages educated by sEVs from RKO‐shSLC8A1 cells significantly reduced the radiosensitivity of RKO cells (Figure S6M–O).

Clinical analysis revealed significantly lower SLC8A1 expression in CRC tissues compared to paired normal tissues (Figure 5M). Notably, IHC analysis demonstrated that SLC8A1 expression was higher in patients achieving pCR after neoadjuvant therapy than in non‐pCR cases (Figure 5N,O). Further analysis in rectal cancer specimens revealed a significant negative correlation between tumor SLC8A1 levels and M2 TAMs infiltration (Figure S6P). Moreover, diminished tumor SLC8A1 expression was associated with significantly poorer OS and DFS (Figure 5P,Q). Together, these clinical data indicate that reduced SLC8A1 expression in CRC is associated with unfavorable treatment response, enhanced M2 TAMs infiltration, and inferior survival outcomes.

Together, these data demonstrate that SLC8A1 deficiency disrupts calcium homeostasis by impairing Ca^2+^ extrusion, resulting in sustained accumulation of radiation‐induced intracellular Ca^2+^. Elevated Ca^2+^ levels subsequently facilitate ANXA2^+^sEV secretion, thereby promoting M2 TAM polarization and establishing a tumor microenvironment that confers radioresistance.

## 4 Discussion

The fundamental challenge in overcoming CRC radioresistance lies in deciphering how radiotherapy dynamically remodels the immunosuppressive TME. Through integrated in vivo modeling and single‐cell transcriptomics, we demonstrate that SLC8A1 deficiency in CRC cells disrupts calcium homeostasis, leading to elevated intracellular Ca^2^^+^ levels. This Ca^2^^+^ surge promotes selective loading of ANXA2 into sEVs. Upon uptaking by TAMs, ANXA2^+^sEVs drive M2 polarization via STAT6 phosphorylation, ultimately establishing therapeutic resistance. Our study provides the first evidence that tumor‐TME crosstalk, mediated by the SLC8A1‐Ca^2^^+^‐ANXA2^+^sEVs‐STAT6 signaling axis, constitutes a unified mechanism underpinning radiotherapy failure in CRC.

This work significantly advances our understanding of TME remodeling by linking intratumoral heterogeneity at the protein level—specifically, the reduced expression of SLC8A1, a calcium regulatory protein whose downregulation leads to dysregulated calcium homeostasis—to immunosuppressive reprogramming. While dysregulated Ca^2^^+^ signaling has been implicated in tumor progression [34], its role in sEV‐mediated immune remodeling during radiotherapy resistance remains largely unexplored. We demonstrate that radiation‐induced intracellular Ca^2^^+^ accumulation acts not merely as a consequence of cellular stress but as a signaling event that governs the selective packaging of functional molecules into sEVs. In particular, ANXA2, a calcium‐dependent phospholipid‐binding protein [32], represents a critical effector connecting intracellular calcium imbalance with extracellular immune modulation. Mechanistically, calcium influx triggers PTP1B‐mediated dephosphorylation of ANXA2 at tyrosine 24 (Y24), converting ANXA2 from a “closed” conformation (N‐terminus associated with the C‐terminal core domain) to an “open” conformation that exposes its C‐terminal core domain, thereby enabling membrane translocation and lipid raft association [32]. Valapala et al. further demonstrated that ANXA2, following its Ca^2^^+^‐dependent recruitment to the plasma membrane, is internalized via lipid raft endocytosis into multivesicular endosomes and ultimately released into the extracellular space as exosomes [35]. Our immune‐electron microscopy data further support the involvement of ANXA2 redistribution toward membrane‐associated structures. Together, these observations suggest that calcium‐regulated membrane trafficking represents an important determinant of selective sEV cargo loading.

The precise molecular machinery linking elevated intracellular Ca^2^^+^ levels to ANXA2 sorting into sEVs remains to be fully defined. Future studies will be required to determine whether specific calcium sensors, membrane domains, or vesicle biogenesis pathways directly coordinate ANXA2 incorporation. Nevertheless, our findings extend the emerging concept that tumor‐derived sEVs function as active regulators of the TME rather than passive carriers of cellular components. sEVs have previously been implicated in pre‐metastatic niche formation in ovarian cancer [36], astrocytes activation in lung cancer [37], and M2 polarization in CRC [38]. Our study further demonstrates that ANXA2^+^sEVs contribute to therapeutic resistance by reshaping macrophage phenotypes through STAT6 activation, consistent with its well‐documented role in macrophage differentiation [31], which also highlight TAM reprogramming as a critical component of radiation adaptation.

Beyond mechanistic insights, our findings have potential clinical implications for predicting and overcoming radioresistance. Low tumor SLC8A1 levels correlate with increased M2‐TAMs infiltration, poor pathological response, and inferior survival in CRC patients, suggesting that SLC8A1 may serve as a candidate marker for identifying tumors with an immune‐suppressive and radioresistant phenotype. Complementing tissue‐based evaluation, plasma ANXA2^+^sEVs offer dynamic liquid biopsy potential due to their circulatory stability and molecular richness [39, 40]. Crucially, the calcium‐dependent loading of ANXA2 into sEVs provides a functional link between tumor ion homeostasis and detectable blood biomarkers. This enables non‐invasive monitoring of radioresistance mechanisms, where co‐detection of low tumor SLC8A1 mRNA and elevated circulating ANXA2^+^sEVs could enhance predictive accuracy. Such integrated profiling aligns with advances in sEV‐based diagnostics, including PD‐L1^+^sEVs for immunotherapy monitoring [41, 42] and KRAS‐mutant sEV DNA for CRC prognosis [43].

Building on these diagnostic advances, therapeutic targeting of the SLC8A1‐calcium axis offers a promising strategy to reverse radioresistance. Calcium channel blockers (CCBs), which are widely used clinically, may correct radiation‐induced calcium dyshomeostasis and suppress pathogenic sEV secretion. This approach is grounded in calcium biology: Dysregulated Ca^2^^+^ fluxes drive therapy resistance by activating immunosuppressive pathways and vesicular trafficking [44], while pharmacologic Ca^2^^+^ flux normalization enhances treatment sensitivity [44]. Clinical evidence further supports CCBs repurposing: Lacidipine reprograms immunosuppressive Tregs in triple‐negative breast cancer through calcium‐dependent metabolic rewiring [45], and CCBs improve postoperative survival in cholangiocarcinoma by disrupting protumorigenic Ca^2^^+^ signaling [46]. Concurrently, strategies to restore SLC8A1 function could enhance Ca^2^^+^ extrusion capacity, given its role in maintaining ion homeostasis [47] and inverse correlation with CRC radioresistance. Notably, our data using BAPTA‐AM demonstrate that calcium chelation disrupts the resistance cascade, validating ion modulation as defined in radioresistance models.

While this study delineates a key mechanism underlying CRC radioresistance, two limitations warrant consideration. First, clinical radiotherapy is frequently combined with chemotherapy or immunotherapy; our focused investigation on radiotherapy alone precludes assessment of potential synergistic or antagonistic effects of multimodal therapies on immune microenvironment remodeling and therapeutic efficacy. Second, although TAMs were identified as central mediators, the tumor microenvironment constitutes a complex ecosystem orchestrated by multicellular networks interacting via cytokine signaling, metabolic crosstalk, and direct cell–cell contacts.

In conclusion, our study establishes that reduced SLC8A1 expression in CRC cells impairs Ca^2^^+^ extrusion, resulting in elevated [Ca^2^^+^]_i_ relative to SLC8A1‐high tumors. This Ca^2^^+^ dysregulation triggers ANXA2 enrichment within tumor‐derived sEVs. Subsequent uptaking of ANXA2^+^sEVs by TAMs drives M2 polarization through STAT6 phosphorylation. These events collectively drive tumor radioresistance. By defining a tumor‐immune regulatory circuit connecting calcium metabolism, extracellular vesicle biology, and macrophage reprogramming, our findings provide mechanistic insight into radiation adaptation and suggest new opportunities for biomarker development and therapeutic intervention in CRC.

## Author Contributions

**Yimin Fang and Jinghan Zhu**: conceptualization, methodology, investigation, formal analysis, and writing. **Qian Xiao, Qichun Wei, and Xiujing He**: investigation, validation, and data curation. **Ping Li, Kai Jiang, Yue Liu, and Yeting Hu**: resources and methodology. **Weiwei Yu, Chuansheng Guo, Sheng Chen, and Jian Zhao**: formal analysis and visualization. **Siqi Dai, Jingsun Wei, Hang Yang, and Tongtong Bu**: investigation and validation. **Kefeng Ding and Haiyan Chen**: supervision, project administration, funding acquisition, and writing.

## Funding

This study was supported by the National Natural Science Foundation of China (Grant No. 82203704 and No. 82373449), the Key R&D Program of Zhejiang (2023C03049), the Noncommunicable Chronic Diseases‐National Science and Technology Major Project (No. 2024ZD0520100) and Zhejiang Provincial Natural Science Foundation of China (Grant Numbers: LMS25H160015).

## Ethics Statement

Animal studies were approved by the Animal Care and Use Committee of the Second Affiliated Hospital, Zhejiang University School of Medicine (Approval Number: 2022‐222).

## Consent

This study was performed in line with the principles of the Declaration of Helsinki. Approval was granted by the Ethics Committee of Jinhua Municipal Central Hospital (Approval Number: 2018R‐154). Informed consent was obtained from all individual participants included in the study.

## Conflicts of Interest

The authors declare no conflicts of interest.

## Supporting information




**Supporting File**: advs77308‐sup‐0001‐SuppMat.docx.

## Data Availability

The data that support the findings of this study are available from the corresponding author upon reasonable request.

## References

[1] P. Bouchard and J. Efron , “Management of Recurrent Rectal Cancer,” Annals of Surgical Oncology 17, no. 5 (2010): 1343–1356, 10.1245/s10434-009-0861-2. 20041351

[2] W.‐J. Mei , X.‐Z. Wang , Y.‐F. Li , et al., “Neoadjuvant Chemotherapy with CAPOX versus Chemoradiation for Locally Advanced Rectal Cancer with Uninvolved Mesorectal Fascia (CONVERT): Initial Results of a Phase III Trial,” Annals of Surgery 277, no. 4 (2023): 557–564, 10.1097/SLA.0000000000005780. PMC9994847 36538627

[3] W. A. Hall , J. Li , Y. N. You , et al., “Prospective Correlation of Magnetic Resonance Tumor Regression Grade with Pathologic Outcomes in Total Neoadjuvant Therapy for Rectal Adenocarcinoma,” Journal of Clinical Oncology 41, no. 29 (2023): 4643–4651, 10.1200/JCO.22.02525. PMC10564288 37478389

[4] R. D. Hofheinz , F. Wenz , S. Post , et al., “Chemoradiotherapy with Capecitabine versus Fluorouracil for Locally Advanced Rectal Cancer: A Randomised, Multicentre, Non‐Inferiority, Phase 3 Trial,” The Lancet Oncology 13, no. 6 (2012): 579–588. 10.1016/S1470-2045(12)70116-X 22503032

[5] R. Sauer , T. Liersch , S. Merkel , et al., “Preoperative versus Postoperative Chemoradiotherapy for Locally Advanced Rectal Cancer: Results of the German CAO/ARO/AIO‐94 Randomized Phase III Trial after a Median Follow‐up of 11 Years,” Journal of Clinical Oncology 30, no. 16 (2012): 1926–1933, 10.1200/JCO.2011.40.1836. 22529255

[6] T. Suwa , M. Kobayashi , J. M. Nam , and H. Harada , “Tumor Microenvironment and Radioresistance,” Experimental & Molecular Medicine 53 (2021): 1029–1035. 10.1038/s12276-021-00640-9 PMC8257724 34135469

[7] Y. Wu , Y. Song , R. Wang , and T. Wang , “Molecular Mechanisms of Tumor Resistance to Radiotherapy,” Molecular Cancer 22, no. 1 (2023): 96, 10.1186/s12943-023-01801-2. PMC10268375 37322433

[8] Y. Komohara , Y. Fujiwara , K. Ohnishi , and M. Takeya , “Tumor‐Associated Macrophages: Potential Therapeutic Targets for Anti‐Cancer Therapy,” Advanced Drug Delivery Reviews 99, no. Pt B (2016): 180–185. 10.1016/j.addr.2015.11.009 26621196

[9] A. Mantovani , P. Allavena , F. Marchesi , and C. Garlanda , “Macrophages as Tools and Targets in Cancer Therapy,” Nature Reviews Drug Discovery 21, no. 11 (2022): 799–820, 10.1038/s41573-022-00520-5. PMC9380983 35974096

[10] K. H. Parker , P. Sinha , L. A. Horn , et al., “HMGB1 Enhances Immune Suppression by Facilitating the Differentiation and Suppressive Activity of Myeloid‐Derived Suppressor Cells,” Cancer Research 74, no. 20 (2014): 5723–5733, 10.1158/0008-5472.CAN-13-2347. PMC4199911 25164013

[11] J. Chen , R. M. McKay , and L. F. Parada , “Malignant Glioma: Lessons from Genomics, Mouse Models, and Stem Cells,” Cell 149, no. 1 (2012): 36–47, 10.1016/j.cell.2012.03.009. PMC3719882 22464322

[12] J. Gu , J. Zhou , Q. Chen , et al., “Tumor Metabolite Lactate Promotes Tumorigenesis by Modulating MOESIN Lactylation and Enhancing TGF‐β Signaling in Regulatory T Cells,” Cell Reports 39, no. 12 (2022): 110986, 10.1016/j.celrep.2022.110986. 35732125

[13] L. van Hooren , S. M. Handgraaf , D. J. Kloosterman , et al., “CD103(+) regulatory T Cells Underlie Resistance to Radio‐Immunotherapy and Impair CD8(+) T Cell Activation in Glioblastoma,” Nature Cancer 4, no. 5 (2023): 665–681. 10.1038/s43018-023-00547-6 PMC10212765 37081259

[14] C. Jiménez‐Cortegana , C. Galassi , V. Klapp , D. I. Gabrilovich , and L. Galluzzi , “Myeloid‐Derived Suppressor Cells and Radiotherapy,” Cancer Immunology Research 10, no. 5 (2022): 545–557, 10.1158/2326-6066.CIR-21-1105. 35426936

[15] P. Sharma , B. A. Siddiqui , S. Anandhan , et al., “The Next Decade of Immune Checkpoint Therapy,” Cancer Discovery 11, no. 4 (2021): 838–857, 10.1158/2159-8290.CD-20-1680. 33811120

[16] C. M. Jackson , A. Pant , W. Dinalankara , et al., “The Cytokine Meteorin‐Like Inhibits Anti‐Tumor CD8(+) T Cell Responses by Disrupting Mitochondrial Function,” Immunity 57, no. 8 (2024): 1864–1877. 10.1016/j.immuni.2024.07.003 PMC11324406 39111315

[17] M. Poggio , T. Hu , C.‐C. Pai , et al., “Suppression of Exosomal PD‐L1 Induces Systemic Anti‐Tumor Immunity and Memory,” Cell 177, no. 2 (2019): 414–427.e13, 10.1016/j.cell.2019.02.016. PMC6499401 30951669

[18] C. Liu , X. Zhou , H. Zeng , et al., “Endoplasmic Reticulum Stress Potentiates the Immunosuppressive Microenvironment in Hepatocellular Carcinoma by Promoting the Release of SNHG6‐Enriched Small Extracellular Vesicles,” Cancer Immunology Research 12, no. 9 (2024): 1184–1201, 10.1158/2326-6066.CIR-23-0469. 38900485

[19] Y. Yin , B. Liu , Y. Cao , et al., “Colorectal Cancer‐Derived Small Extracellular Vesicles Promote Tumor Immune Evasion by Upregulating PD‐L1 Expression in Tumor‐Associated Macrophages,” Advanced Science 9, no. 9 (2022): 2102620. 10.1002/advs.202102620 PMC8948581 35356153

[20] L. Shen , H. Huang , Z. Wei , et al., “Integrated Transcriptomics, Proteomics, and Functional Analysis to Characterize the Tissue‐Specific Small Extracellular Vesicle Network of Breast Cancer,” MedComm 4, no. 6 (2023): 433, 10.1002/mco2.433. PMC10694390 38053815

[21] J. A. Welsh , D. C. I. Goberdhan , L. O'Driscoll , et al., “Minimal Information for Studies of Extracellular Vesicles (MISEV2023): From Basic to Advanced Approaches,” Journal of Extracellular Vesicles 13, no. 2 (2024): 12404, 10.1002/jev2.12404. PMC10850029 38326288

[22] E. Daguenet , S. Louati , A. S. Wozny , et al., “Radiation‐Induced Bystander and Abscopal Effects: Important Lessons from Preclinical Models,” British Journal of Cancer 123, no. 3 (2020): 339–348, 10.1038/s41416-020-0942-3. PMC7403362 32581341

[23] H. Chen , Y. Fang , S. Dai , et al., “Characterization and Proteomic Analysis of Plasma‐Derived Small Extracellular Vesicles in Locally Advanced Rectal Cancer Patients,” Cellular Oncology 47, no. 5 (2024): 1995–2009, 10.1007/s13402-024-00983-1. PMC12974077 39162991

[24] K. Jiang , H. Chen , Y. Fang , et al., “Exosomal ANGPTL1 Attenuates Colorectal Cancer Liver Metastasis by Regulating Kupffer Cell Secretion Pattern and Impeding MMP9 Induced Vascular Leakiness,” Journal of Experimental & Clinical Cancer Research: CR 40, no. 1 (2021): 21, 10.1186/s13046-020-01816-3. PMC7792106 33413536

[25] E. Gargiulo , E. Viry , P. E. Morande , et al., “Extracellular Vesicle Secretion by Leukemia Cells in Vivo Promotes CLL Progression by Hampering Antitumor T‐Cell Responses,” Blood Cancer Discovery 4, no. 1 (2023): 54–77, 10.1158/2643-3230.BCD-22-0029. PMC9816815 36108149

[26] W. Zhong , Z. Xiao , Z. Qin , et al., “Tumor‐Derived Small Extracellular Vesicles Inhibit the Efficacy of CAR T Cells against Solid Tumors,” Cancer Research 83, no. 16 (2023): 2790–2806, 10.1158/0008-5472.CAN-22-2220. PMC10524031 37115855

[27] M. Zhao , J. Xu , S. Zhong , et al., “Expression Profiles and Potential Functions of Circular RNAs in Extracellular Vesicles Isolated from Radioresistant Glioma Cells,” Oncology Reports 41, no. 3 (2019): 1893–1900. 10.3892/or.2019.6972 30664179

[28] Y. Fang , H. Chen , Y. Liu , et al., “NUPR1 Promotes Radioresistance in Colorectal Cancer Cells by Inhibiting Ferroptosis,” Journal of Cellular and Molecular Medicine 29, no. 7 (2025): 70519, 10.1111/jcmm.70519. PMC11965884 40176685

[29] H. Chen , Q. Xiao , Y. Hu , et al., “ANGPTL1 attenuates Colorectal Cancer Metastasis by Up‐Regulating microRNA‐138,” Journal of Experimental & Clinical Cancer Research 36, no. 1 (2017): 78, 10.1186/s13046-017-0548-7. PMC5467265 28606130

[30] K. E. de Visser and J. A. Joyce , “The Evolving Tumor Microenvironment: from Cancer Initiation to Metastatic Outgrowth,” Cancer Cell 41, no. 3 (2023): 374–403. 10.1016/j.ccell.2023.02.016 36917948

[31] T. Lawrence and G. Natoli , “Transcriptional Regulation of Macrophage Polarization: Enabling Diversity with Identity,” Nature Reviews Immunology 11, no. 11 (2011): 750–761, 10.1038/nri3088. 22025054

[32] C. Zhang , T. Zhou , Z. Chen , et al., “Coupling of Integrin α5 to Annexin A2 by Flow Drives Endothelial Activation,” Circulation Research 127, no. 8 (2020): 1074–1090. 10.1161/CIRCRESAHA.120.316857 32673515

[33] S. Yoodee , T. Malaitad , S. Plumworasawat , and V. Thongboonkerd , “E53, E96, D162, E247 and D322 in Ca(^2+^)‐Binding Domains of Annexin A2 Are Essential for Regulating Intracellular [Ca(^2+^)] and Crystal Adhesion to Renal Cells via ERK1/2 and JNK Signaling Pathways,” Archives of Biochemistry and Biophysics 769 (2025): 110410, 10.1016/j.abb.2025.110410. 40189002

[34] D. Khananshvili , “The SLC8 Gene family of Sodium–calcium Exchangers (NCX)—Structure, Function, and Regulation in Health and Disease,” Molecular Aspects of Medicine 34, no. 2‐3 (2013): 220–235, 10.1016/j.mam.2012.07.003. 23506867

[35] M. Valapala and J. K. Vishwanatha , “Lipid Raft Endocytosis and Exosomal Transport Facilitate Extracellular Trafficking of Annexin A2,” Journal of Biological Chemistry 286, no. 35 (2011): 30911–30925, 10.1074/jbc.M111.271155. PMC3162451 21737841

[36] L. Gao , X. Nie , R. Gou , et al., “Exosomal ANXA2 Derived from Ovarian Cancer Cells Regulates Epithelial‐Mesenchymal Plasticity of human Peritoneal Mesothelial Cells,” Journal of Cellular and Molecular Medicine 25, no. 23 (2021): 10916–10929, 10.1111/jcmm.16983. PMC8642686 34725902

[37] M. He , G. Xie , F. Shen , and X. Li , “Activation of Astrocytes by ANXA2‐Derived Extracellular Vesicles from Lung Cancer Cells Affects Aggressiveness, Immunotherapy Response and Microenvironment of Lung Cancer,” Heliyon 10, no. 6 (2024): e27729, 10.1016/j.heliyon.2024.e27729. PMC10965531 38545147

[38] L. Zhou , J. Li , M. Liao , Q. Zhang , and M. Yang , “LncRNA MIR155HG Induces M2 Macrophage Polarization and Drug Resistance of Colorectal Cancer Cells by Regulating ANXA2,” Cancer Immunology, Immunotherapy 71, no. 5 (2022): 1075–1091. 10.1007/s00262-021-03055-7 PMC10991596 34562123

[39] G. Wang , J. Li , L. Bojmar , et al., “Tumour Extracellular Vesicles and Particles Induce Liver Metabolic Dysfunction,” Nature 618, no. 7964 (2023): 374–382, 10.1038/s41586-023-06114-4. PMC10330936 37225988

[40] D. Yu , Y. Li , M. Wang , et al., “Exosomes as a New Frontier of Cancer Liquid Biopsy,” Molecular Cancer 21, no. 1 (2022): 56, 10.1186/s12943-022-01509-9. PMC8855550 35180868

[41] S. M. Morrissey and J. Yan , “Exosomal PD‐L1: Roles in Tumor Progression and Immunotherapy,” Trends in Cancer 6, no. 7 (2020): 550–558. 10.1016/j.trecan.2020.03.002 PMC7330176 32610067

[42] S. Serratì , M. Guida , R. Di Fonte , et al., “Circulating Extracellular Vesicles Expressing PD1 and PD‐L1 Predict Response and Mediate Resistance to Checkpoint Inhibitors Immunotherapy in Metastatic Melanoma,” Molecular Cancer 21, no. 1 (2022): 20, 10.1186/s12943-021-01490-9. PMC8764806 35042524

[43] D. Lucchetti , I. V. Zurlo , F. Colella , et al., “Mutational Status of Plasma Exosomal KRAS Predicts Outcome in Patients with Metastatic Colorectal Cancer,” Scientific Reports 11, no. 1 (2021): 22686, 10.1038/s41598-021-01668-7. PMC8608842 34811396

[44] S. Marchi , C. Giorgi , L. Galluzzi , and P. Pinton , “(Ca2+) Fluxes and Cancer,” Molecular Cell 78, no. 6 (2020): 1055–1069, 10.1016/j.molcel.2020.04.017. 32559424

[45] Y. Sheng , C. Qiao , Z. Zhang , et al., “Calcium Channel Blocker Lacidipine Promotes Antitumor Immunity by Reprogramming Tryptophan Metabolism,” Advanced Science 12, no. 3 (2025): 2409310. 10.1002/advs.202409310 PMC11744582 39585774

[46] K. Kodama , T. Kawaoka , M. Kosaka , et al., “Calcium Channel Blockers Improve the Prognosis of Patients with Intrahepatic Cholangiocarcinoma after Resection,” Journal of Gastroenterology 57, no. 9 (2022): 676–683, 10.1007/s00535-022-01887-3. 35849192

[47] Q. S. Liao , Q. Du , J. Lou , J. Y. Xu , and R. Xie , “Roles of Na + /Ca 2+ Exchanger 1 in Digestive System Physiology and Pathophysiology,” World Journal of Gastroenterology 25, no. 3 (2019): 287–299, 10.3748/wjg.v25.i3.287. PMC6343099 30686898

