# Supplementary material for: Low SLC8A1 in Colorectal Cancer Dictates Radiotherapy Resistance Through Calcium‐Dependent ANXA2+sEVs Driving M2 Polarization of TAMs
Source: Adv Sci (Weinh). 2026 Aug 27:e77308. Online ahead of print. doi: 10.1002/advs.77308 (PMC13521182; doi:10.1002/advs.77308)
Supplement: Supplementary file 1 [file ADVS-9999-e77308-s001.docx]

**Figure S1**

**(A-C)** In vitro radiation response in MC38/MC38-RR/MC38-RRvt cells showed reduced viability (CCK-8, 0-8 Gy, day7; A), suppressed lipid peroxidation (6 Gy flow cytometry; B), and decreased apoptosis (6 Gy flow cytometry; C) in radioresistant lines. **(D-H)** scRNA-seq analysis revealed immune dynamics: experimental design (D), CD45⁺ immune cell clustering (E) with subset proportions (F), T cell subclustering (G) and subset distribution (H). **(I)** IHC confirmed reduced F4/80⁺ infiltration after clodronate. **(J-L)** mIHC analysis of paired pre-/post-neoadjuvant therapy specimens. (J) Representative images (pCR/non-pCR; DAPI: blue, CD68: green, CD163: red), (K) CD68⁺CD163⁺ cell quantification in pCR cohort (pre- vs post-therapy), (L) CD68⁺CD163⁺ cell quantification in non-pCR cohort (pre- vs post-therapy). **(M-N)** MSK cohort validation confirmed worse OS (M) and DFS (N) with high M2-TAMs infiltration. Statistics: One-way ANOVA (multiple groups), unpaired t-test (two groups), log-rank test (survival); **P*<0.05, ***P*<0.01, ****P*<0.001, *****P*<0.0001.

**Figure S2**

**(A-C)** sEVs isolation and characterization from MC38, MC38+IR, MC38-RR, and MC38-RR+IR cells: (A) WB of sEVs markers (ALIX, TSG101, CD9, Calnexin), (B) Transmission electron microscopy analysis of sEVs, (C) The number of particles per milliliter and median diameter was determined by dynamic light scattering analysis. **(D-I)** Polarization capacity of sEV-containing fractions. BMDMs educated for 24h with components from irradiated (6 Gy) or non-irradiated cells: CM groups: M2 proportion (flow cytometry; D) and Cd206/Arg1/Tgf-β mRNA (qPCR; E); sEVs groups: M2 proportion (F) and gene expression (G); sEV-depleted CM groups: M2 proportion (H) and gene expression (I). **(J-K)** sEVs uptake dynamics. (D) In vitro internalization: PKH67-labeled sEVs (10 μg/ml) in BMDMs at 4h (fluorescence microscopy); (E) In vivo tracking: Intratumoral PKH67-sEVs (10 μg/20µL qod×3) in MC38 tumors; mIF (DAPI: blue, PKH67: green, F4/80: red). Statistics: Unpaired t-test (two groups). Significance: **P* < 0.05, ***P* < 0.01, ****P* < 0.001, *****P* < 0.0001.

**Figure S3**

**(A-E)** BMDMs co-cultured 24h with ± irradiated (6 Gy) MC38-RR cells ± GW4869 (20 μM): (A-B) M2 polarization (flow cytometry), (C-E) Cd206/Arg1/Tgf-β mRNA (qPCR). **(F)** Rab27a KD validation (WB). **(G-K)** BMDMs co-cultured 24h with MC38, MC38-RR-shNC, or MC38-RR-shRab27a cells (± 6 Gy): (G-H) M2 polarization quantified by flow cytometry, (I-K) Corresponding Cd206, Arg1, Tgf-β mRNA levels (qPCR). **(L-N)** MC38-RR-shRab27a vs control tumors receiving radiotherapy (8 Gy×2 at 100 mm³): Growth curves (L), endpoint sizes (M), masses (N). Statistics: One-way ANOVA (multiple groups), unpaired t-test (two groups). Significance: **P*<0.05, ***P*<0.01, ****P*<0.001, *****P*<0.0001*.*

**Figure S4**

**(A-B)** Proteomic profiling identified 18 common differentially expressed proteins in sEVs from: 1) MC38-RR ± radiotherapy (60 proteins), 2) post-radiotherapy MC38-RR vs MC38 (72 proteins), A: Venn diagram; B: Volcano plot. **(C)** ANXA2, ERO1A and VCP in sEVs from MC38 and MC38-RR cells ± radiotherapy (6 Gy, WB). **(D)** Anxa2 knockdown efficiency (WB). **(E)** ANXA2 in sEVs from MC38-RR-shNC and MC38-RR-shAnxa2 cells ± radiotherapy (6 Gy, WB). **(F)** WB detection of ANXA2 levels in MC38‑RR‑IR‑sEVs after treatment with proteinase K ± Triton X‑100. **(G)** KEGG enrichment analysis revealed JAK-STAT pathway activation in MC38-RR-IR-sEV-educated versus MC38-IR-sEV-educated BMDMs. **(H)** Heatmap of differentially expressed STAT6 target genes across experimental groups. **(I-M)** BMDMs co-treated 24h with sEVs (10μg/ml) ± 6 Gy IR ± AS: (I-J) M2 polarization (flow cytometry), (K-M) Cd206/Arg1/Tgf-β mRNA (qPCR). **(N)** STAT6 knockdown efficiency in BMDMs (WB). **(O-S)** STAT6‑knockdown BMDMs were educated with MC38‑RR‑IR‑sEVs (10μg/ml) for 24 h. (O-P) M2 polarization by flow cytometry; (Q-S) qPCR of Cd206, Arg1, Tgf‑β mRNA (n=3). Statistics: One‑way ANOVA (multiple groups) or unpaired t‑test (two groups). Significance: ***P*<0.01, ****P*<0.001, *****P*<0.0001*.*

**Figure S5**

**(A-C)** Cal treatment (500 nM) elevated intracellular Ca²⁺ in MC38 cells (flow cytometry: A-B; fluorescence microscopy: C). **(D)** Cal treatment did not increase sEV secretion (BCA assay). **(E-F)** BA chelation attenuated radiation-induced Ca²⁺ elevation (flow cytometry: E) and ANXA2-sEV enrichment (WB: F)**. (G)** WB validation of ANXA2 knockout and reconstitution with wild‑type or E96A mutant in MC38‑RR cells. **(H)** Flow cytometric analysis of intracellular Ca²⁺ levels post‑IR (6 Gy) in MC38‑RR‑NC, MC38‑RR‑ANXA2 KO, MC38‑RR‑KO‑wt, and MC38‑RR‑KO‑E96A cells (n=3). **(I-M)** BMDMs were educated for 24 h with sEVs (10μg/ml) from irradiated cells described above. (I-J) M2 polarization by flow cytometry; (K-M) qPCR of Arg1, Cd206, Tgf‑β mRNA (n=3). **(N)** ANXA2 elevation in sEVs from Cal-treated (500 nM, 24h) vs untreated RKO cells. **(O-P)** THP-1 cells co-treated 24h with RKO ± Cal-sEV (10μg/ml): M2 polarization quantified by CD206, CD163 mRNA levels (qPCR), n=3. **(Q-R)** Clonogenic survival of RKO cells co-cultured with THP-1 cells educated by RKO ± Cal-sEV (10μg/ml) for 24 hours, followed by IR (6 Gy). The survival data were normalized to those of unirradiated cells, n=3. **(S)** CCK-8 assay of RKO cells co-cultured with THP-1 cells educated by RKO ± Cal-sEV (10μg/ml) for 24 hours, followed by IR (6 Gy), n=5. Statistics: One-way ANOVA (multiple groups), unpaired t-test (two groups). Significance: ***P*<0.01, ****P*<0.001, *****P*<0.0001.

**Figure S6**

**(A-D)** Transcriptional dysregulation of Ca²⁺ regulators in MC38-RR vs MC38 (qPCR of Atp2b4/Cacna1a/Cacna1h/Itpr2). **(E)** Slc8a1 knockdown efficiency in MC38 cells (WB). **(F)** Slc8a1 knockdown elevated radiation-induced Ca²⁺. **(G-H)** mIHC of irradiated tumors from MC38-shNC and MC38-shSlc8a1: (G) Representative images (DAPI: nuclei; F4/80: green; CD206: red). (H) Quantified F4/80⁺CD206⁺ cells/HPF (n=6). **(I)** Slc8a1 knockdown efficiency in RKO cells (WB). **(J)** ANXA2 in sEVs from RKO-shNC and RKO-shSLC8A1 cells ± IR (6 Gy, WB). **(K-L)** THP-1 cells co-treated 24h with RKO-shNC/RKO-shSLC8A1 ± IR (6Gy): M2 polarization quantified by CD206, CD163 mRNA levels (qPCR), n=3. **(M-N)** Clonogenic survival of RKO cells co-cultured with THP-1 cells educated by RKO-shNC/RKO-shSLC8A1+IR-sEV for 24 hours, followed by IR (6 Gy). The survival data were normalized to those of unirradiated cells, n=3. **(O)** CCK-8 assay of RKO cells co-cultured with THP-1 cells educated by RKO-shNC/RKO-shSLC8A1+IR-sEV for 24 hours, followed by IR (6 Gy), n=5. **(P)** Negative correlation between SLC8A1 expression and M2 TAMs signature (Pearson correlation). Statistics: One-way ANOVA (multiple groups), Pearson (correlation), unpaired t-test (two groups). Significance: **P*<0.05, ***P*<0.01, ****P*<0.001, *****P*<0.0001.

**Supplementary Table 1. Antibodies used for Western blotting**

| **Antibodies** | **Catalog No.** | **Clone No.** | **Vendors** | **Working dilution** |
| --- | --- | --- | --- | --- |
| Anti-ALIX | 92880 | E6P9B | CST | 1:1000 |
| Anti-TSG101 | 72312 | E6V1X | CST | 1:1000 |
| Anti- CD9 | 98327 | E8L5J | CST | 1:1000 |
| Anti- CALNEXIN | 10427-2-AP | / | Proteintech | 1:1000 |
| Anti-SLC8A1 | 28447-1-AP | / | Proteintech | 1:1000 |
| Anti- STAT6 | 51073-1-AP | / | Proteintech | 1:1000 |
| Anti-STAT6(phospho Y641) | ab263947 | EPR22599-78 | Abcam | 1:1000 |
| Anti-ANXA2 | ET1704-49 | JA42-30 | Huabio | 1:1000 |
| Anti-STAT3 | ET1607-38 | SY24-08 | Huabio | 1:1000 |
| Anti- STAT3(phospho Y705) | ET1603-40 | SZ43-01 | Huabio | 1:1000 |
| Anti- RAB27A | 66058-1-Ig | 1C4B8 | Proteintech | 1:1000 |
| Anti-GAPDH | HA721136 | PD00-07 | Huabio | 1:5000 |
| Anti-TUBLIN | 14555-1-AP | / | Proteintech | 1:5000 |
| Anti-VCP | ET1703-56 | JM11-15 | Huabio | 1:1000 |
| Anti-ERO1A | HA722818 | PSH07-30 | Huabio | 1:1000 |

**Supplementary Table 2. Primers for qRT-PCR**

| **gene** |  |  | Sequence **(5'-3')** |
| --- | --- | --- | --- |
| m-Cd206 | Forward  Reverse |  | GTCAGAACAGACTGCGTGGA AGGGATCGCCTGTTTTCCAG |
| m-Arg1 | Forward  Reverse |  | CTTGCGAGACGTAGACCCTG TGAGTTCCGAAGCAAGCCAA |
| m-Tgf-β | Forward  Reverse |  | CATCCATGACATGAACCGGC  GAAGTTGGCATGGTAGCCCT |
| m-Il1rn | Forward  Reverse |  | TAGACATGGTGCCTATTGACCT  TCGTGACTATAAGGGGCTCTTC |
| m-Retnla | Forward  Reverse |  | CCAATCCAGCTAACTATCCCTCC  ACCCAGTAGCAGTCATCCCA |
| m-Chil3 | Forward  Reverse |  | CAGGTCTGGCAATTCTTCTGAA  GTCTTGCTCATGTGTGTAAGTGA |
| m-Il10ra | Forward  Reverse |  | GCCCTTCCTATGTGTGGTTTG  TTGAGTTTCCGTACTGTTTGAGG |
| m-Slc8a1 | Forward  Reverse |  | GCTGGTCGGTTTCTCATCCA  TGGGCAAGATCACCCCTTTC |
| m-Cacna1h | Forward  Reverse |  | ATGCTTGGGAACGTGCTTCTT  GTCTGGTAGTATGGCCGCAA |
| m-Itpr2 | Forward  Reverse |  | GAGCTCTTGGACAACCCAGG  CTAGTGGCCGAAGTTGCTGA |
| m-Il10ra | Forward  Reverse |  | GCCCTTCCTATGTGTGGTTTG  TTGAGTTTCCGTACTGTTTGAGG |
| m-Atp2b4 | Forward  Reverse |  | TACATCTCCTCCTGTGGGCA  AACGATTTGCACTTGGTGGC |
| m-Cacna1a | Forward  Reverse |  | TCTCTGGGCCGATACACTGA  CGGAACTACTGCCCTGTCTG |
| m-β-actin | Forward  Reverse |  | GGCTGTATTCCCCTCCATCG  CCAGTTGGTAACAATGCCATGT |
| CD163 | Forward |  | TTTGTCAACTTGAGTCCCTTCAC |
|  | Reverse |  | TCCCGCTACACTTGTTTTCAC |
| CD206 | Forward |  | TCCGGGTGCTGTTCTCCTA |
|  | Reverse |  | CCAGTCTGTTTTTGATGGCACT |
| β-actin | Forward |  | CATGTACGTTGCTATCCAGGC |
|  | Reverse |  | CTCCTTAATGTCACGCACGAT |

**Supplementary Table 3. Clinicopathological Characteristics of pCR Group versus non-pCR Group** **in the Central Cohort**

|  | **pCR Group** | **non-pCR Group** |  |
| --- | --- | --- | --- |
| **Patients（N = 55）** | 14 | 41 |  |
| **Age（medium-range）** | 61.5(34-83) | 62(39-79) | *P* = 0.5714 |
| **Gender** |  |  | *P* = 0.5696 |
| Male | 11 | 29 |  |
| Female | 3 | 12 |  |
| **Degree of tumor differentiation** |  |  | *P* = 0.6839 |
| Poorly differentiated | 1 | 2 |  |
| Moderately differentiated | 11 | 36 |  |
| Well differentiated | 2 | 3 |  |
| **T Stage** |  |  | *P* = 0.5660 |
| 2 | 2 | 2 |  |
| 3 | 10 | 27 |  |
| 4 | 3 | 10 |  |
| **N Stage** |  |  | *P* = 0.2736 |
| 0 | 7 | 11 |  |
| 1 | 5 | 20 |  |
| 2 | 2 | 10 |  |
| **Stage** |  |  | *P* = 0.2625 |
| Ⅱ | 6 | 11 |  |
| Ⅲ | 8 | 30 |  |

**Supplementary Table 4. Clinicopathological Characteristics of high M2 TAMs infiltration Group versus low M2 TAMs infiltration Group in the MSK public database**

|  | **high M2 Group** | **low M2 Group** |  |
| --- | --- | --- | --- |
| **Patients（n = 84）** | 9 | 75 |  |
| **Age（medium-range）** | 61 (32-71) | 53(26-78) | *P* = 0.3461 |
| **Gender** |  |  | *P* = 0.9596 |
| Male | 5 | 41 |  |
| Female | 4 | 34 |  |
| **Clinical LN** |  |  | *P* = 0.2240 |
| Positive | 8 | 62 |  |
| Negative | 1 | 13 |  |
| **Tumor Size** |  |  | *P* = 0.0572 |
| <4cm | 4 | 22 |  |
| 4-8cm | 3 | 45 |  |
| >8cm | 2 | 3 |  |
| Unknow | 0 | 5 |  |

**Supplementary Table 5. Overlapping upregulated sEV proteins in two independent comparisons**

| Gene | MC38-RR+IR vs MC38-RR  logFC | MC38-RR+IR vs MC38-RR adj.P.Val | MC38-RR+IR vs MC38+IR logFC | MC38-RR+IR vs MC38+IR adj.P.Val |
| --- | --- | --- | --- | --- |
| **Ero1a** | **1.903** | **7.57e-05** | **1.540** | **0.000263** |
| Anxa4 | 1.589 | 0.000535 | 0.534 | 0.0411 |
| **Anxa2** | **1.352** | **0.00259** | **0.908** | **0.0187** |
| Paics | 1.166 | 0.0016 | 0.462 | 0.0345 |
| Yars1 | 1.059 | 0.00167 | 0.931 | 0.00185 |
| **Vcp** | **0.889** | **0.0289** | **1.189** | **0.00135** |
| Aprt | 0.602 | 0.0305 | 0.947 | 0.00263 |
| Ndrg1 | 0.481 | 0.0141 | 0.813 | 0.0025 |
| Tes | 0.810 | 0.00957 | 0.797 | 0.0135 |
| Dstn | 0.697 | 0.00329 | 0.778 | 0.00316 |
| Hpcal1 | 0.407 | 0.0196 | 0.731 | 0.000947 |
| Mif | 0.684 | 0.00195 | 0.672 | 0.00577 |
| Kpna2 | 0.678 | 0.00107 | 0.479 | 0.00868 |
| Tpi1 | 0.576 | 0.00937 | 0.536 | 0.00908 |
| Tpp2 | 0.646 | 0.00142 | 0.514 | 0.0233 |
| Hsp90b1 | 0.643 | 0.0119 | 0.468 | 0.0361 |
| Csnk2a1 | 0.451 | 0.0241 | 0.579 | 0.00757 |
| Lgals1 | 0.438 | 0.0276 | 1.229 | 9.75e-05 |

**Supplementary Table 6. 33 calcium signaling‑related DEGs identified between MC38‑RR and parental MC38 cells**

| Gene | logFC | adj.P.Val |
| --- | --- | --- |
| Bdkrb1 | 3.497905766 | 5.62e-10 |
| Adcy3 | 2.498596699 | 4.56e-09 |
| Plcd4 | 1.969877391 | 1.08e-08 |
| Bdkrb2 | 1.915966375 | 6.35e-06 |
| Mylk2 | 1.795086697 | 0.030086658 |
| Cacna1a | 1.517340197 | 0.000108018 |
| Itpr2 | 1.448465667 | 8.62e-30 |
| Pdgfrb | 1.36072329 | 4.26e-09 |
| Adcy7 | 1.202214683 | 1.23e-16 |
| Egfr | -1.474290365 | 7.78e-15 |
| Nos1 | -1.085828102 | 1.25e-24 |
| Pdgfra | -1.441898651 | 1.10e-49 |
| Ednra | -1.829522494 | 6.87e-06 |
| Fgfr2 | -1.351757853 | 2.39E-22 |
| Ntrk2 | -1.439793598 | 0.004612205 |
| Itpka | -1.500005664 | 0.001004027 |
| Pdgfc | -1.761266117 | 1.10e-14 |
| Fgf21 | -1.951959786 | 1.06e-07 |
| Atp2b4 | -2.24331826 | 2.09e-05 |
| Camk2a | -2.302446915 | 1.74e-07 |
| Pde1a | -2.372901244 | 5.99e-43 |
| Ryr1 | -2.478047297 | 0.018829332 |
| Ryr2 | -2.813231488 | 0.029234348 |
| Fgf9 | -2.892765496 | 2.29e-09 |
| Pde1c | -3.766336901 | 2.48e-79 |
| Cacna1h | -3.937478915 | 0.047170595 |
| Mst1 | -3.998120267 | 1.25e-08 |
| Adcy2 | -4.192432401 | 0.001272019 |
| Adra1a | -4.558267634 | 0.006724603 |
| Ptk2b | -4.913400894 | 1.99e-58 |
| Nos3 | -6.599912842 | 0.024252749 |
| Chrm1 | -6.832890014 | 0.016603837 |
| Slc8a1 | -7.532798646 | 3.44e-07 |
